# Self-patterning of human stem cells into post-implantation lineages

**DOI:** 10.1038/s41586-023-06354-4

**Published:** 2023-06-27

**Authors:** Monique Pedroza, Seher Ipek Gassaloglu, Nicolas Dias, Liangwen Zhong, Tien-Chi Jason Hou, Helene Kretzmer, Zachary D. Smith, Berna Sozen

**Affiliations:** 1 Department of Genetics, Yale School of Medicine, Yale University, New Haven, CT USA; 2 Yale Stem Cell Center, Yale University, New Haven, CT USA; 3 Department of Genome Regulation, Max Planck Institute for Molecular Genetics, Berlin, Germany; 4 Department of Obstetrics, Gynecology and Reproductive Sciences, Yale School of Medicine, Yale University, New Haven, CT USA

**Keywords:** Embryology, Embryonic stem cells, Cell signalling, Differentiation

## Abstract

Investigating human development is a substantial scientific challenge due to the technical and ethical limitations of working with embryonic samples. In the face of these difficulties, stem cells have provided an alternative to experimentally model inaccessible stages of human development in vitro^1–13^. Here we show that human pluripotent stem cells can be triggered to self-organize into three-dimensional structures that recapitulate some key spatiotemporal events of early human post-implantation embryonic development. Our system reproducibly captures spontaneous differentiation and co-development of embryonic epiblast-like and extra-embryonic hypoblast-like lineages, establishes key signalling hubs with secreted modulators and undergoes symmetry breaking-like events. Single-cell transcriptomics confirms differentiation into diverse cell states of the perigastrulating human embryo^14,15^ without establishing placental cell types, including signatures of post-implantation epiblast, amniotic ectoderm, primitive streak, mesoderm, early extra-embryonic endoderm, as well as initial yolk sac induction. Collectively, our system captures key features of human embryonic development spanning from Carnegie stage^16^ 4–7, offering a reproducible, tractable and scalable experimental platform to understand the basic cellular and molecular mechanisms that underlie human development, including new opportunities to dissect congenital pathologies with high throughput.

## Abstract (web summary)

Human pluripotent stem cells can be triggered to self-organize into structures recapitulating early human post-implantation embryonic development.

## Main

The entire human body emerges from a cluster of pluripotent embryonic cells that form in the first week of life, and their proper development depends on support from the peripheral extra-embryonic tissues to transport nutrients and supply patterning-associated morphogens. Limited accessibility and ethical restrictions have substantially impeded our ability to investigate human development, creating gaps between what can be learned from model organisms with species-specific differences in developmental timing, differentiation and spatial geometry. Recently, increasingly sophisticated in vitro stem cell models of human embryogenesis have provided more tractable opportunities to understand the complex biological processes that govern fetal viability and long-term health^1–13^. Although valuable models for post-implantation human development exist^1–7^, most still lack extra-embryonic lineages and cell types despite their integral role in establishing the human body plan in vivo. Here we present a new integrative system that captures essential tissue interactions between the early embryonic epiblast and extra-embryonic hypoblast lineages from an initially pluripotent population and spans critical periods of early human post-implantation development. This system facilitates the efficient self-organization into complex 3D structures that proceed through the initial patterning of amniotic-like and primitive streak-like lineages. This strategy serves as a robust experimental method to investigate multiple critical and human-specific features of development across spatial and temporal scales, including how diverse and differentiating cell types interface to support synchronized patterning of post-implantation human embryo development.

## Reproducible 3D assembly of human pluripotent stem cells

**Fig. 1:**
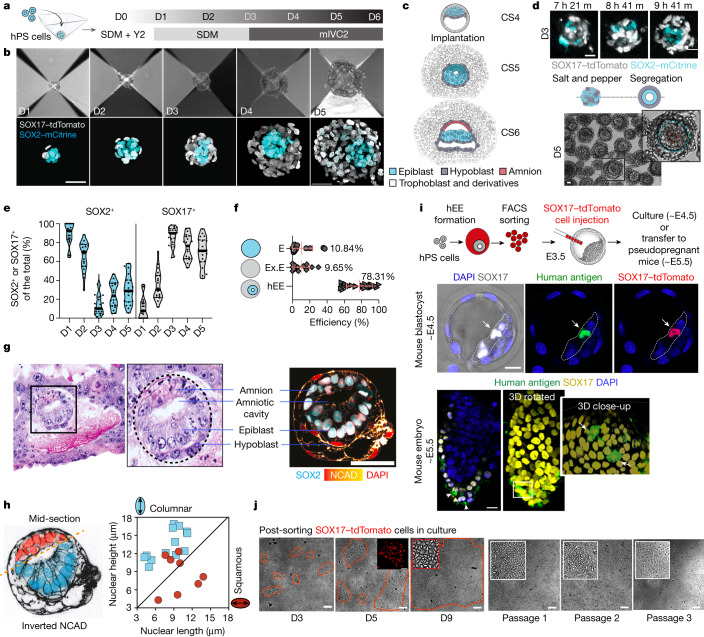
hEEs reproducibly model post-implantation-like lineage bifurcation. **a**, Schematic of hEE generation. SDM, spontaneous differentiation medium. SDM, spontaneous differentiation media. Y2, Y27632. mIVC2, modified in vitro culture 2 media. **b**, Time-course development of hEEs. Scale bar, 50 µm. *n* = 11, 6 and 7 independent experiments from RUES2, H9 and ESI017 hPS cells, respectively. **c**, Schematic of human embryo development at the indicated Carnegie stages (CS). **d**, Sampled frames from a timelapse movie of hEE organization (top). *n* = 30 structures; *n* = 3 independent experiments. Scale bar, 20 µm. Phase-contrast image of D5 hEEs (bottom). The inset image highlights the inner cavity (red circle), epiblast-like (blue circle) and hypoblast-like (white circle) compartments. *n* ≥ 30 independent experiments. Scale bar, 20 µm. **e**, Percentage of SOX2^+^ and SOX17^+^ cells in hEEs. *n* = 20 structures per timepoint. Each dot represents one structure. The plot shows the median (thick solid line) and quartiles (thin dotted line). **f**, hEE efficiency versus aggregates comprising a single compartment (embryonic-like (E) and extra-embryonic-like (Ex.E)). Plots show mean ± s.d. *n* = 1,764 structures from D4 and D5. *n* = 24 independent experiments for hEE and 13 independent experiments for embryonic-like only and extra-embryonic-like only (for the noted genetic background variation, see Supplementary Table 2). **g**, Histological section of a CS5b stage in vivo human embryo (left; obtained from the Virtual Human Embryo project) compared with an in vitro hEE at D5 (right). Scale bar, 50 µm. *n* = 9 independent experiments. **h**, Same structure as panel **g** but with inverted and enhanced N-cadherin (NCAD) intensity for better clarity (left). An ozone graph of nuclear length and height of presumptive amnion-like (red) and epiblast-like (blue) cells is also shown (right). **i**, Chimeric integration of hEE-derived SOX17–tdTomato cells into primitive (top) or visceral endoderm (bottom) of mouse E4.5 blastocysts or E5.5 embryos. *n* = 15/54 blastocysts and 4/10 E5.5 embryos. *n* = 2 independent experiments. Scale bars, 20 µm. The arrows indicate the successful integration of human SOX17-TdTomato cells into the mouse primitive or visceral endoderm. **j**, Expansion of SOX17–tdTomato cells in 2D culture, which were sorted from D3 or D4 hEEs. The red lines outline cell colonies, which are also shown in the zoomed-in images. *n* = 3 independent experiments. Scale bars, 200 µm. Illustrations in **a**,**c**,**i**, credit: A.L. Cox. The embryo section in **g**, courtesy of the Virtual Human Embryo. Source Data

**Extended Data Fig. 1:**
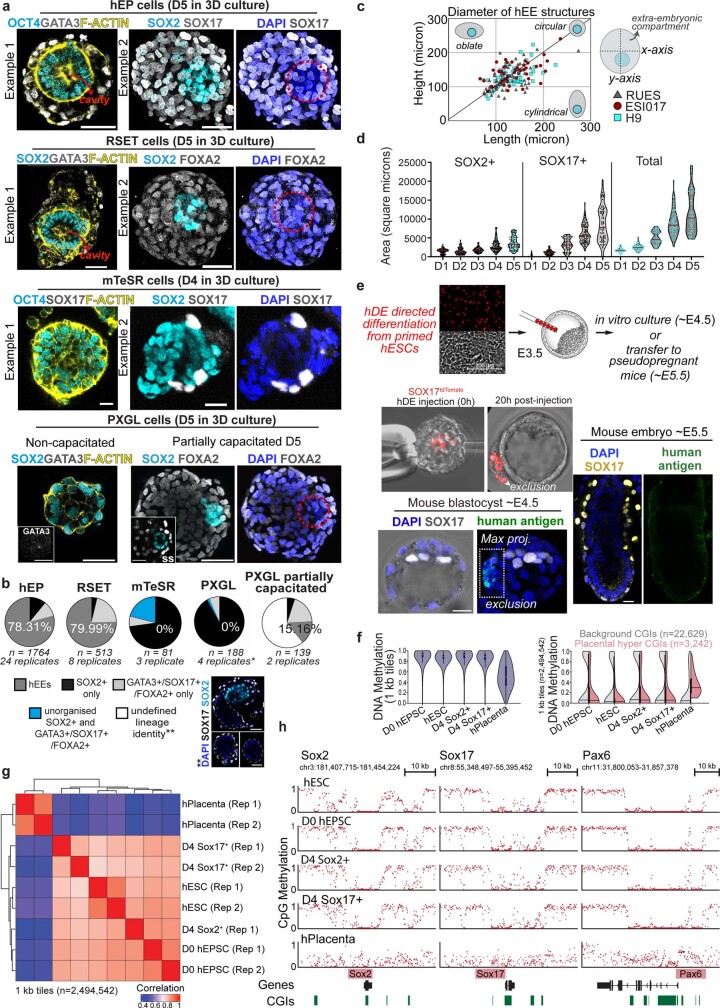
Evaluating stem cell pluripotency states by their potential to form human extra-embryoids. (**a**) hEEs generated from hEP, RSeT, mTeSR, or PXGL hPSCs. Scale bar, 50 μm. N values and independent experiments are presented in panel b. (**b**) Pie charts displaying the yield of multicellular aggregates in panel a. Percentages correspond to hEE formation. *PXGL (non-capacitated) = 4 independent experiments total. Note that 2 experiments failed to aggregate. mTeSR = 3 independent experiments. Note that 2 experiments failed to aggregate. Insert images show examples of undefined lineage identity(**). Scale bars, 50 μm. (**c**) Diameter of hEEs generated from RUES2 (N = 50), ESI017 (N = 50), H9 (N = 50) hPSC lines. 10 independent experiments. (**d**) Area of SOX2+, SOX17+, and total regions of hEEs from D1 through D5 of culture. D1 (N = 109), D2 (N = 86); D3 (N = 73); D4 (N = 97), D5 (N = 79). Each dot represents one analysed structure. The plot shows the median (solid, red line) and quartiles (red, dotted line). 3 independent experiments. (**e**) Chimeric integration strategy to inject directed-differentiated definitive endoderm from primed hPSCs to mouse embryos. Representative E4.5 blastocyst and E5.5 embryo with SOX17-tdTomato+ cell (also marked by human antigen in green) were excluded from the Sox17+ primitive or visceral endoderm layer (white or yellow). 0/49 embryos, 4 independent experiments. Scale bar, 20 μm. (**f**) Violin plots showing the global methylation levels (left) and split violin plots for CpG islands (right) of hEE compared to hESCs and human placenta^24^. White circles and horizontal bars represent the median, boxes the interquartile range (IQR), and whiskers 1.5xIQR. (**g**) Hierarchical clustering of 1-kb tiles across hEE replicate samples compared to hESC and placental samples. (**h**) Genome browser tracks of three developmental loci in hEE samples as they compare to hESCs and human Placenta (as proxies for embryonic and extraembryonic landscapes, respectively). Illustrations in **e**, credit: A. L. Cox. Source Data

The ability to model hidden aspects of human development in vitro brings new opportunities to advance human-specific biology and biomedical research. Here we used human pluripotent stem (hPS) cells maintained under conditions that support intermediate pluripotency states between ground and primed pluripotency (RSeT^17^, EP^18^ and partially capacitated PXGL^19^; see Methods). These hPS cells were aggregated in 3D and exposed to ‘spontaneous differentiation medium’ (SDM, see Methods) formulated for this study that provides minimal growth factor support (Fig. 1a). Within 48 h, the hPS cells self-organized into structures with bifurcation into distinct SOX2^+^ and SOX17^+^, FOXA2^+^ or GATA3^+^ cell types (Fig. 1b and Extended Data Fig. 1a,b). This initial lineage segregation resembled the epiblast–hypoblast patterning of implanting-stage human embryos (Fig. 1b–d). Conversely, hPS cells maintained under conventional primed pluripotency conditions (mTeSR) did not yield organized structures (Extended Data Fig. 1a,b).

After 72 h, we exchanged medium into a ‘modified in vitro culture 2 medium’ (see Methods) previously reported for human post-implantation embryos^15^ (Fig. 1a). Under this optimized condition, aggregates acquired a spheroid morphology comprising an acentrically positioned inner epiblast-like and an outer extra-embryonic hypoblast-like compartment (Fig. 1b–e and Extended Data Fig. 1c,d). Compared with ex vivo cultured human embryos^15^, the rate of cell proliferation was similar for human epiblast-like cells but somewhat higher for the hypoblast-like lineage (Fig. 1e, Extended Data Fig. 1d and Supplementary Table 1).

hPS cells bifurcate into structured SOX2^+^ and SOX17^+^ compartments consistently, with an average efficiency of 78.31% of aggregates showing these features across experimental trials and cell lines (Fig. 1f and Supplementary Table 2). Of note, hPS cells maintained under naive pluripotency conditions (PXGL^20^) could also form organized structures, but only after the formative pluripotency transition and with a lower overall efficiency (‘partial capacitation’^19^; see Methods; Extended Data Fig. 1a,b). By 120 h, the SOX2^+^ inner compartment appeared to self-organize into an epithelial cyst with asymmetrically patterned columnar cells along one pole and squamous-like cells at the other, a morphology that resembles the bipolar CS5b human embryo (day 9 post-fertilization) (Fig. 1g,h). As this model appears to capture extra-embryonic tissue-level interactions, we refer to these structures as human extra-embryoids (hEEs).

To functionally validate the extra-embryonic endoderm (hypoblast)-like cells in hEEs, we generated structures from SOX17–tdTomato reporter hPS cells and isolated SOX17–tdTomato^+^ cells after 72–96 h of specification to test their chimeric competency when injected into early mouse blastocysts (Fig. 1i). hEE-derived SOX17–tdTomato^+^ cells successfully integrated into the primitive endoderm lineage in 27.77% of cases, indicating a functional contribution to the extra-embryonic lineage, whereas definitive endoderm-differentiated cells derived from primed hPS cells^21^ did not (Fig. 1i and Extended Data Fig. 1e). Chimeric embryos transferred into pseudopregnant surrogate mice confirmed the persistence of human cells within the extra-embryonic visceral endoderm layer, although their viability was largely compromised in what probably reflects a xenogeneic barrier (Fig. 1i). Of note, hEE-isolated SOX17–tdTomato^+^ cells could also expand when transferred into culture conditions previously reported to exclusively support the extra-embryonic endoderm^22^. Although we cannot rule out the potential presence of non-expanding definitive endoderm cells^22^, these results further support hEEs as an in vitro model for early extra-embryonic endoderm development (Fig. 1j).

To molecularly explore the epigenome of hEE SOX2^+^ and SOX17^+^ cells, we also performed whole-genome bisulfite sequencing to see whether these cells acquire unique epigenomic features of these lineages that are acquired during mammalian implantation^23,24^. Ultimately, undifferentiated human embryonic pluripotent stem (hEPS) cells and SOX2^+^ and SOX17^+^ hEE cells exhibited very similar DNA methylation landscapes, including global hypermethylation and constitutive hypomethylation of CpG islands found at developmental gene promoters (Extended Data Fig. 1f–h). These findings suggest that the formation of hEEs from hEPS cells does not include global epigenomic reprogramming that might occur in vivo, although further understanding of the epigenetic dynamics of the human hypoblast lineage are only beginning to be explored^25^.

**Extended Data Fig. 2:**
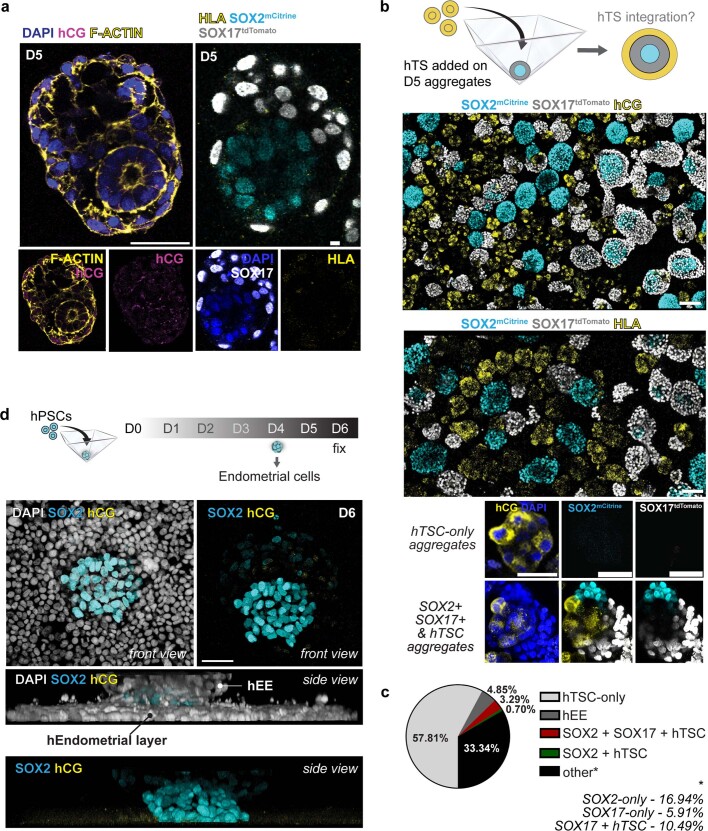
Human extra-embryoid do not initiate spontaneous trophoblast differentiation. (**a**) Representative hEEs at D5 are negative for trophectoderm markers hCG (magenta) and HLA (yellow). Scale bar, 50 μm (left) and 20 μm (right). N = 82 structures from D3 to D6. 3 independent experiments. (**b**) **Top**: Schematic of strategy to incorporate human trophoblast stem cells (hTSCs, marked by HLA and hCG in yellow) into hEEs at D5. **Bottom**: Wide-field tile scans show that TSCs and hEEs form independent aggregates and do not mix after extended co-culture. Scale bars, 20 μm. N = 554 structures, 2 independent experiments. (**c**) Pie chart of the proportion of aggregates in panel b. N = 554 structures, 2 independent experiments. (**d**) **Top**: Schematic of strategy to promote trophoblast-like differentiation: D4 hEE structures were transferred from 3D wells onto a monolayer of endometrial cells and imaged at D6. **Bottom**: 3D projection of SOX2+ (cyan) and hCG (yellow) in a D6 structure from a front and side view. Structures (white line) interacted with the endometrial layer (indicated by a black line) from front and side views but were negative for hCG (yellow). N = 42 structures, 3 independent experiments. Scale bar, 50 μm. Illustrationsin **b**,**d**, credit: A. L. Cox. Source Data

Finally, we interrogated hEEs for their ability to differentiate into trophoblast as neither hCG nor HLA expression was observed in our model (Extended Data Fig. 2a). hEEs also did not integrate with human trophoblast stem cells^26^ in co-culture, but instead formed isolated hCG^+^HLA^+^ aggregates that compromised hEE formation (Extended Data Fig. 2b,c). Similarly, co-culture with human epithelial endometrial cells did not stimulate trophoblast differentiation through reciprocal signalling with hEEs (Extended Data Fig. 2d).

Overall, these morphological observations demonstrate that this 3D platform appears to efficiently capture the plasticity of an intermediate pluripotent state in humans that is able to self-organize into structures that resemble the cellular composition of the human embryonic disc.

## Validation of major lineages by single-cell RNA sequencing

**Fig. 2:**
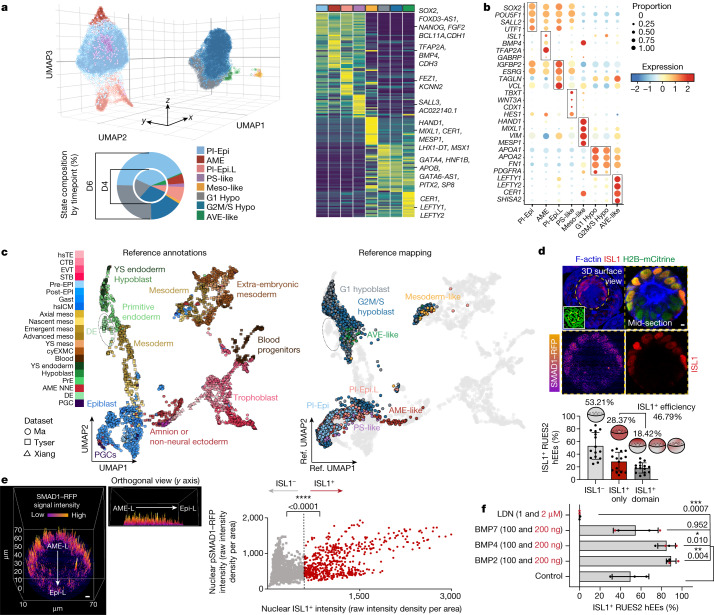
The emergence of perigastrulation lineages in hEEs. **a**, 3D uniform manifold approximation and projection (UMAP) plot (top), pie charts (bottom) and gene expression heatmap (right) of D4 and D6 structures. *n* = 18,042 total cells. G1 Hypo, growth 1 hypoblast-like; G2M/S Hypo, growth 2 mitosis/synthesis hypoblast-like; meso-like, mesoderm-like; PS-like, primitive streak-like. **b**, Dot plot of mean marker gene expression level. **c**, Integrated reference of three studies of primate development (left). See Methods and Supplementary Notes. Projection of hEE scRNA-seq data (query) onto in vivo reference is also shown (right). hEE cells are annotated as above. Reference cells are in grey to represent the background distribution of primate states. For the in vivo reference lineage abbreviations, refer to Extended Data Fig. 3d and Supplementary Table 3. **d**, hEEs generated from SMAD1–RFP hPS cells (top). Scale bar, 20 µm. The inner compartment (dotted lines, schematics) ISL1 phenotype frequency is also shown (bottom). Each dot represents the percentage of structures per tile scan. The plot shows mean ± s.d. *n* = 346 structures from 2 independent experiments specific to the RUES2 background are presented in the graph. *n* = 13 experiments total across different genetic backgrounds. **e**, SMAD1–RFP 3D surface intensity plot (same structure as shown in panel **d**) (left). Scale bar, 20 µm. A scatter plot of nuclear SMAD1–RFP fluorescence intensity in ISL1^−^ and ISL1^+^ cells. *n* = 1,677 cells in 3 representative structures from 2 independent experiments. Two-tailed unpaired, parametric *t*-test with Welsch’s correction. *P* values are displayed in the figure. **f**, Percentage of ISL1^+^ structures (RUES2 background). D4 and D5 control (*n* = 489), BMP2 (*n* = 461), BMP4 (*n* = 405), BMP7 (*n* = 391) and LDN (*n* = 521). Three or four independent experiments per group. Post-hoc Dunnett’s multiple comparison test, one-way ANOVA. *P* values are displayed in the figure. Mean ± s.d. are shown. Source Data

**Extended Data Fig. 3:**
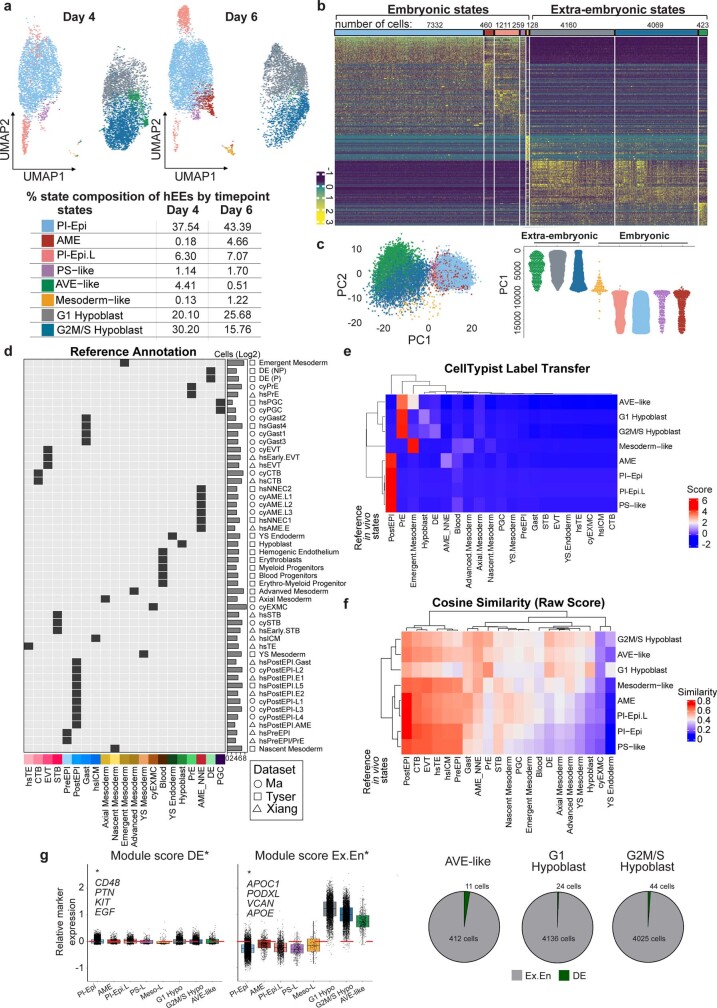
Embryonic- and extra-embryonic-like cell types resemble human embryo lineages. (**a**) **Top**: 3D UMAP plot shown in Fig. 2a with D4 and D6 time points separated in 2D. **Bottom**: Percentages for each cell state are included for each time point. For lineage abbreviations, refer to Fig. 2a. (**b**) Gene expression within D4 and D6 structures including the number of cells per cell state. The colour scale represents row-scaled marker gene expression level. (**c**) PCA plot (left) and PC1 rank (right) of cells assigned to embryonic- and extra-embryonic-like lineages in hEEs. For lineage abbreviations, see Fig. 2a. (**d**) Table of merged state annotations to connect our three *in vivo* reference samples (see Methods). Bars represent the number of cells in each original label (log2 transformed) to highlight the difficulty in fine-resolution cell state assignments using machine learning. For *in vivo* reference lineage abbreviations please refer to Supplementary Table 3. Data sets are indicated as Xiang et al.^15^ (triangle), Ma et al.^28^ (circle), and Tyser et al.^14^ (square). (**e**) Heatmap of mean CellTypist decision scores between hEE cells and each *in vivo* reference state (see Methods). Scores were averaged for each hEE cell type and row-scaled to highlight the degree to which they match each respective reference label. Rows and columns are clustered using Euclidean distance. (**f**) Cosine similarity heatmap comparing average gene expression between hEE and reference states (see Methods and Supplementary Notes). (**g**) **Left**: Box plots that show the per-cell module score for Definitive Endoderm (DE) and Extra-embryonic Endoderm (Ex.En) associated transcripts. For lineage abbreviations, refer to the legend for Fig. 2a. Each dot represents a single cell. Boxplot shows interquartile range as bars, median as black midline, and quartile ranges as vertical lines. N = 18,042 cells from 4 independent differentiation experiments. **Right**: The pie charts represent the proportion of cells within extra-embryonic clusters that are positive for Definitive Endoderm (DE) or Extra-embryonic Endoderm (Ex.En) associated transcripts. N numbers for sample size is shown in the charts, 4 independent differentiation experiments.

To examine the developmental complexity of hEEs, we applied single-cell RNA sequencing (scRNA-seq) at two progressive timepoints, D4 and D6, and resolved eight distinct transcriptional clusters. Our states clearly fall within one of two major lineages, roughly characterized as post-implantation epiblast like (PI-Epi; *SOX2*, *POU5F1*, *SALL2* and *UTF1*) and hypoblast like (*APOA1*, *APOA2*, *FN1* and *PDGFRA*) (Fig. 2a,b and Extended Data Fig. 3a–c). Of note, the temporal resolution of our hEE dataset allowed us to make several intriguing observations from D4 to D6 that suggest a coordinated differentiation sequence. Within the embryonic compartment, we observed progressive formation of amnion-like (*ISL1*, *BMP4*, *TFAP2A* and *GABRP*) as well as a primitive streak-like (*TBXT*, *WNT3A*, *CDX1* and *HES1*) cellular states at D4, with the emergence of an embryonic mesoderm-like state (*HAND1*, *MIXL1*, *MESP1* and *VIM*) at D6. We also observed cells that resemble a later differentiated embryonic state (PI-Epi.L, referring to ‘late’) that expresses several early ectodermal or early amnion markers such as *IGFBP2*, *ESRG*, *TAGLN* and *VCL*^14^ (Fig. 2a,b and Extended Data Fig. 3a,b). By contrast, the hypoblast-like lineage comprises three clusters, although two clusters appear to represent the same general hypoblast state within different cell cycle phases. A distinct and transient third state within this lineage was characterized by significantly high expression of NODAL, BMP and FGF antagonists (for example, *CER1*, *LEFTY1*, *LEFTY12* and *SHISA2*) that resemble key molecular hallmarks of the anterior visceral endoderm (AVE-like) in mouse embryos^27^ (Fig. 2a,b and Extended Data Fig. 3a,b).

To confirm the similarity between our hEE transcriptional states and their in vivo counterparts, we integrated our dataset with recent major breakthrough datasets that span their expected developmental window, including in vitro-cultured human perigastrulation embryos^15^, a CS7 human gastrulating embryo implanted in utero^14^ and in vitro-cultured gastrulating embryos from cynomolgus monkeys^28^. Projection of our integrated dataset onto a reference uniform manifold approximation and projection (UMAP) model confirmed the overall segregation between embryonic-associated, hypoblast-associated and trophoblast-associated regions, with hEE data well positioned exclusively within embryonic (PI-Epi, PI-Epi.L, amnion-like and mesoderm-like cells) and hypoblast (Hypo- and AVE-like cells) states (Fig. 2c, Extended Data Fig. 3d, Supplementary Table 3 and Supplementary Notes 1–4). As expected, hEE states remained highly distinct from those descriptive of primate trophoblast cells (Fig. 2c). We further validated these associations using CellTypist^29^, which trains a machine learning model on the integrated in vivo reference to score each hEE cell. Overall, these efforts confirmed the major bifurcation of embryonic and extra-embryonic lineages, as well as detection of mesoderm-like and amnion-like cell states as they are described in vivo (Extended Data Fig. 3e,f). Finally, we found that our SOX17^+^ cells were enriched for empirically determined extra-embryonic endoderm markers compared with definitive endoderm markers^22^, consistent with their assignment as hypoblast-like states (Extended Data Fig. 3g). Together, our transcriptomic analyses support our annotation and assignment of hEEs to peri-implantation or post-implantation developmental stages of the hypoblast-like and epiblast-like development.

## Emergence of late amnion-like cells

**Extended Data Fig. 4:**
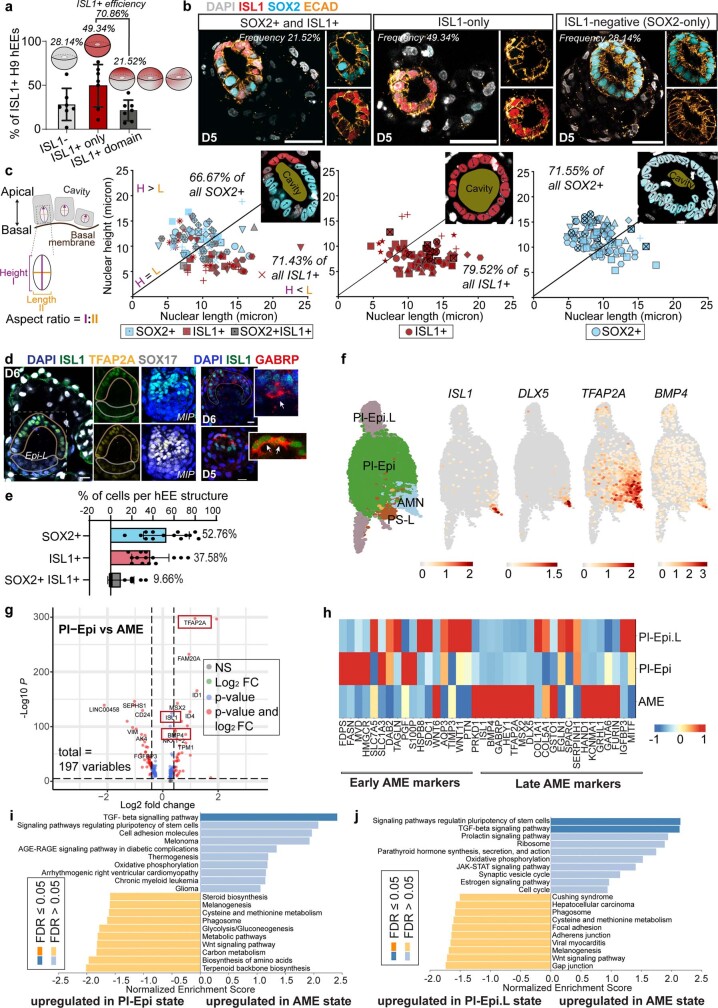
The hEE amnion-like lineage exhibits squamous morphology and expresses key primate amnion markers. (**a**) Percentage of ISL1 phenotype frequencies in H9 hEEs. The plot shows mean ± SD. N = 297 structures, 7 independent experiments. (**b**) Representative structures in panel a. Scale bars, 50μm. (**c**) **Left**: Schematic of height and length aspect ratio quantification to determine nuclear shape. **Right**: Ozone graph of nuclear length and height of SOX2+ (blue), ISL1+ (red), and SOX2+ ISL1+ (grey) cells from the hEE phenotypes introduced in A. Each symbol represents one cell. Each shape corresponds to an individual structure. Each graph includes ≥10 aggregates and >80 cells for 3–5 independent experiments. Schematic shows transparent nuclear masks atop of SOX2+ ISL1+, ISL1+, and SOX2+ cells in D5 structures with the central cavity highlighted in yellow and arrows highlighting the nuclear orientation. (**d**) Co-staining of ISL1 with TFAP2A (left) or GABRP (right) in representative D5 and D6 hEE structures. 5 independent experiments. Scale bar, 20 μm. (**e**) Bar graph of the proportion of SOX2+, ISL1+, and SOX2+ ISL1+ (double positive) cells. N = 14 structures, 4 independent experiments. Each dot represents the percentage of each cell type in each structure. Plots show mean ± SD. (**f**) The expression level of primate amnion markers (*ISL1*, *DLX5*, *TFAP2A*, and *BMP4*) in hEEs. PI-Epi – Post-implantation Epiblast-like (green); AME – Amnion-like (light blue); PI-Epi. L, Postimplantation Epiblast-Late-like (light purple); PS-like, Primitive Streak-like (brown). Colour scale reflects normalised transcript counts. (**g**) Volcano plot of differentially expressed genes between Amnion-like and Postimplantation Epiblast-like populations in the hEE, including primate amnion markers (*TFAP2A*, *ISL1*, and *BMP4*) upregulated in Amnion-like population (red boxes). Two-tailed, non-parametric Wilcoxon Rank-sum test was applied. (**h**) Expression of early and late amniotic wave markers identified from Rostovskaya et al.^36^ in D4 and D6 hEE structures. Expression matrices are row-scaled, colour scale reflects log_2_ fold change from centre value. (**i**-**j**) Gene Set Enrichment Analysis of upregulated KEGG pathways in PI-Epi vs AME (i) and PI-Epi.L vs AME (j) states in hEEs. Source Data

We next investigated the specification of epiblast-derived cell populations, specifically focusing on amnion patterning, which forms before gastrulation in primates. Previous studies of non-human primates and human stem cells suggest that amnion induction is dependent on BMP^30–33^, but many functional and molecular features of this lineage remain unclear. Morphologically, hEEs frequently contained ISL1^+^ cells (a marker of primate amnion^34^) by D4 to D6, with efficiency ranging from 46.79% to 70.86% depending on the genetic background (Fig. 2d, Extended Data Fig. 4a and Supplementary Table 2). We found that 18.42–21.52% of all hEEs exhibited spatially localized ISL1^+^ populations, consistent with recent observations in non-human primates^34^ (Fig. 2d and Extended Data Fig. 4a). A subpopulation of aggregates (28.37–49.34% of all hEEs) have inner compartments exclusively composed of ISL1^+^ cells, suggesting possible artefacts in hEE patterning that could result from signalling imbalances and/or mechanical cues^35^. ISL1 expression correlates with TFAP2A and seemingly weak GABRP expression, as well as with expected morphological changes from the columnar epithelium typical of pre-specified SOX2^+^ epiblast-like cells to a more squamous-like shape (Extended Data Fig. 4b–e).

Primate amniogenesis has recently been described as occurring through two temporally and spatially independent waves^36^. When we explored the overall transcriptional signature of our hEE amnion state, we found higher expression of previously associated primate late-stage amnion marker genes (enriched for *ISL1*, *DLX5*, *TFAP2A*, *BMP4*, *PRKD1*, *GABRP*, *HEY1*, *MSX2* and *GSTO1*) than early-stage amnion marker genes (*FASN*, *MVD*, *SDC1*, *S100P* and *SLC1A3*; Extended Data Fig. 4f–h). By contrast, the Pl-Epi.L cluster expressed genes associated with amnion progression^36^, including *COL1A1*, *COL5A1*, *EGLN1*, *SPARC* and *IGFBP3* (Extended Data Fig. 4h). In addition, a subset of endoderm cells showed *ISL1* expression, similar to non-human primate embryos^34^ (Fig. 2b). *BMP4* expression was highest in the amnion cluster compared with other epiblast-derived cell populations (Extended Data Fig. 4f–h), which might indicate that the functional relationship between BMP4 and ISL1 found in other primates^34^ extends to humans. Differential expression analysis between individual epiblast-like cell states confirms that genes associated with TGFβ signalling are substantial within the amnion state, including major BMP signalling components such as *ID1–ID4* and *BMP4* (Extended Data Fig. 4i,j).

**Extended Data Fig. 5:**
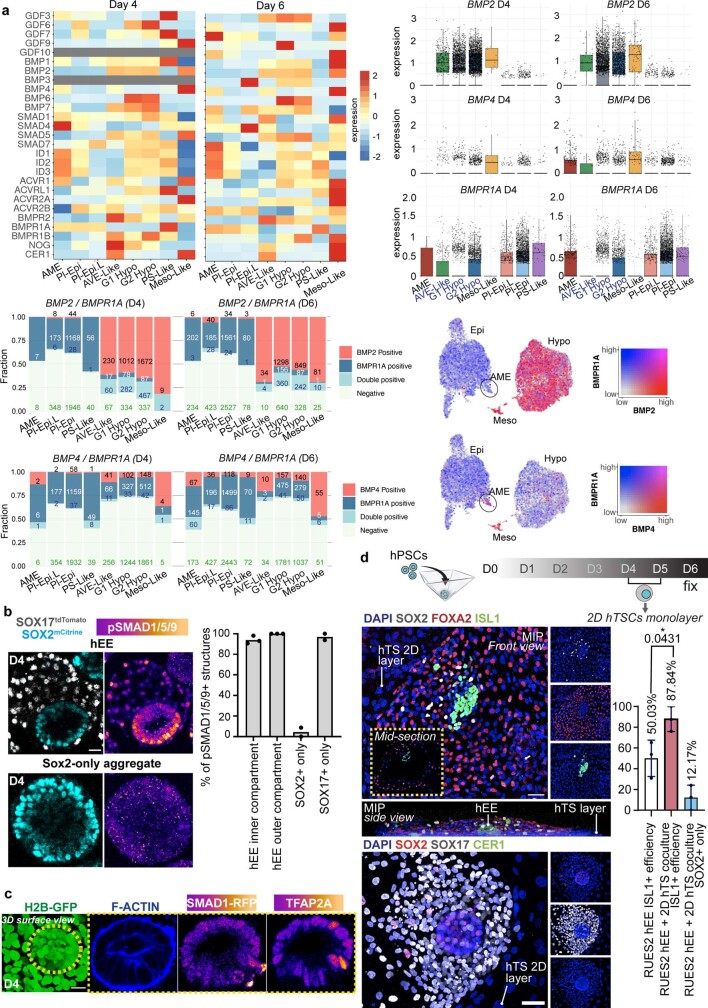
The expression of key BMP signalling components in the human extra-embryoid lineages. (**a**) **Top-left**: Expression of BMP signalling factors in embryonic- and extra-embryonic-like lineages in D4 and D6 hEEs. Expression matrices are row-scaled, color scale reflects relative expression. **Top-right**: Normalised *BMP2, BMPR1A*, and *BMP4* transcript counts are shown in boxplots for each state by day. Each dot represents a single cell. Boxplot shows interquartile range as bars, median as black midline and quartile ranges as vertical lines. N = 18,042 cells from 4 independent differentiation experiments. **Bottom-left**: Barplot data showing fractions of cells per state or day that express *BMP2* or *BMP4* alone or in combination with *BMPR1A*. The numbers on the bar plot represent the N of cells analysed. The number are color-coded accordingly: *BMP2* or *4* positive = black, *BMPR1A* positive = white, double positive = blue, and negative = green. Pooled from 4 independent differentiation experiments. **Bottom-right**: UMAPs show differential co-expression plots of *BMP2* or *BMP4* with the receptor *BMPR1A*. **(b) Left**: SOX2-mCitrine (cyan), SOX17-tdTomato (grey), and pSMAD1/5/9 (Fire intensity gradient on Fiji; purple low, yellow high) in a D4 structure. **Right**: Percentages of pSMAD1;5;9+ structures in SOX17+ and SOX2+ only structures and hEE outer and inner compartments. hEE (N = 241), SOX2-only (N = 28), SOX17-only (N = 34). 3 independent experiments for hEE and 2 independent experiments for SOX2-only and SOX17-only. Scale bar, 20μm. (**c**) 3D surface view and single-sections of H2B-GFP, F-ACTIN, SMAD1-RFP (Fire gradient on Fiji; purple low, yellow high), and TFAP2A (Fire gradient on Fiji) in D4 hEE. N = 66 structures, 2 independent experiments. Scale bar, 20 μm. (**d**) **Top**: Schematic of hEE and 2D human trophoblast stem cells (hTSCs) in co-culture. **Bottom-left**: Max intensity projections of ISL1, SOX2, FOXA2, SOX17, CER1 and DAPI in D6 hEEs on hTS 2D cell layer from a front and side view. Yellow, dotted square encloses the inner compartment of hEE. **Bottom-right**: Percentage of structures with SOX2+ or ISL1+ only efficiencies in inner compartments of control hEEs (N = 489) and hEEs co-cultured on hTSCs (N = 38 structures). 3 independent experiments each. Two-sided unpaired, parametric t-test with Welsch’s correction. The figure shows P values. Plots show mean ± SD. Scale bar, 50 μm. Illustrations in **d**, credit: A. L. Cox. Source Data

More generally, all epiblast-like-derived subclusters, including our initial epiblast-like population, express a gene encoding a BMP receptor, *BMPR1A*, whereas hypoblast-like subclusters showed differential expression for the BMP transducer gene *BMP2* and other components (Extended Data Fig. 5a). This suggested the possibility that the hypoblast may serve a supportive role in amnion-like induction in hEEs. To further explore BMP signalling dynamics, we analysed the phosphorylation status of SMAD transducers, which reflect functional TGFβ superfamily signal transduction. Consistent with our transcriptional results, we first found that hEE structures exhibit universal BMP-associated phosphorylation of SMAD1, SMAD5 and SMAD9 (pSMAD1/5/9) nuclear signal within both the inner embryonic and the surrounding hypoblast-like layers, whereas aggregates that failed to specify hypoblast-like layers lacked the pSMAD1/5/9 signal (Extended Data Fig. 5b). We further utilized the transgenic live reporter SMAD1–RFP;H2B–mCitrine hPS cell line to track and confirm SMAD1 nuclear localization within amnion-like cells, indicating a BMP gradient within the inner compartment of hEEs (Fig. 2e and Extended Data Fig. 5c). To confirm the functional relationship between BMP signalling and amnion induction, we treated hEEs with either the BMP antagonist LDN193189 (LDN) or exogenous BMP2 and BMP4, which significantly repressed or induced ISL1 expression, respectively (Fig. 2f).

According to our transcriptional data, hypoblast-like cells express BMP2, not BMP4 (Extended Data Fig. 5a). The latter is expected to be expressed by trophoblasts, the presumed initial source of proximal BMP signalling in vivo. To test whether either lineage could support amnion induction, we cultured hEEs from D4 to D5 on top of a human trophoblast stem cell-covered surface, which readily induced ‘all-ISL^+^’ inner compartments (Extended Data Fig. 5d). Collectively, these results demonstrate that the hEE system effectively differentiates into temporally organized expression domains that resemble the amnion. Our transcriptional data suggest that this population represents a late amnion state^36^, which is expected to begin early in gastrulation, depends on a BMP-dependent mechanism and appears to rely on the surrounding hypoblast-like lineage in hEEs.

## Lineage co-evolution drives morphogenesis

**Fig. 3:**
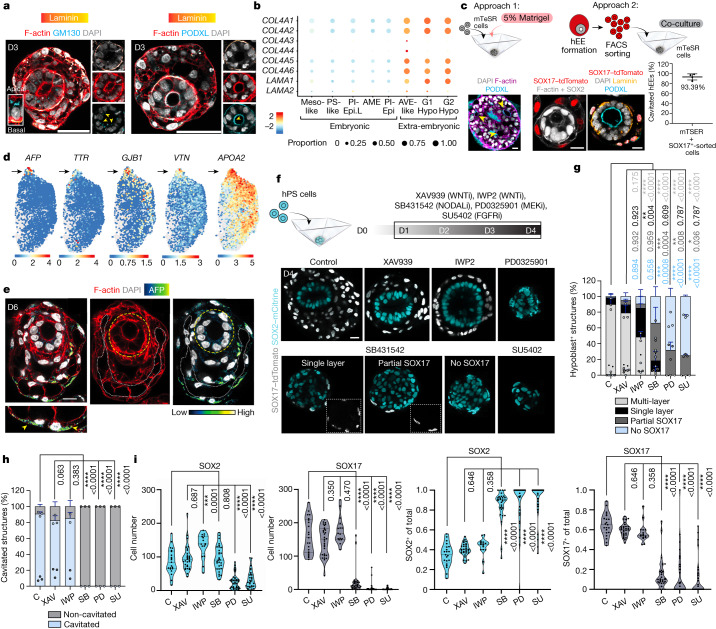
TGFβ and FGF signalling stabilizes hypoblast-like specification and embryo-like morphogenesis. **a**, GM130 (*n* = 169) and PODXL (*n* = 156) immunofluorescence within D3 hEEs. Four independent experiments each. Scale bars, 50 μm. Yellow arrowheads indicate the expression of apical markers. **b**, Dot plot of basement membrane gene expression in hEEs. For lineage abbreviations, refer to Fig. 2a. **c**, Schematic of mTeSR hPS cell aggregation strategies (top), their corresponding D4 structures (bottom left) and the cavitated hEE efficiency (bottom right). The yellow arrowheads indicate multiple cavities. Plots show mean ± s.d. *n* = 439 structures. Data are from four technical replicates and two independent experiments. Scale bars, 20 μm. **d**, UMAP plots of yolk sac endoderm genes within the D6 hypoblast-like cluster. **e**, AFP^+^ staining (arrowheads). The yellow and white dashed lines indicate epiblast-like and yolk sac-like compartment patterning, respectively. *n* = 15 structures over 2 independent experiments. Scale bar, 20 μm. **f**, Inhibitor treatment strategy (top; see Methods) and resulting phenotypes (bottom). The *n* numbers are indicated in panel **g**. Scale bar, 20 μm. **g**, Percentage of structures that specify hypoblast-like lineage. Control (C; *n* = 220), XAV939 (XAV; *n* = 101), IWP2 (IWP; *n* = 132; 2 experiments), SB431542 (SB; *n* = 125), PD0325901 (PD; *n* = 66) and SU5402 (SU; *n* = 109). The plot shows mean ± s.d. Data are from three independent experiments. Post-hoc Dunnett’s multiple comparison test, one-way ANOVA. *P* values are shown in the figure. Each dot represents an independent experiment. **h**, Cavitation efficiency per treatment. Control (*n* = 140), XAV (*n* = 95), IWP (*n* = 118; 2 experiments), SB (*n* = 79), PD (*n* = 66) and SU (*n* = 109). Data are from three independent experiments, unless otherwise indicated. The plot shows mean ± s.d. The same test as panel **g** was applied. *P* values are shown in the figure. Each dot represents an independent experiment. **i**, Number of SOX2^+^ or SOX17^+^ cells per structure and their percentage after each treatment. Each dot represents one structure. Control (*n* = 21), XAV (*n* = 34), IWP (*n* = 16), SB (*n* = 30), PD (*n* = 35) and SU (*n* = 30). Data are from two or more independent experiments. The plot shows the median (thick solid line) and quartiles (thin dotted line). The same test as panel **g** was applied. *P* values are shown in the figure. Illustrations in **c**,**f**, credit: A.L. Cox. Source Data

**Extended Data Fig. 6:**
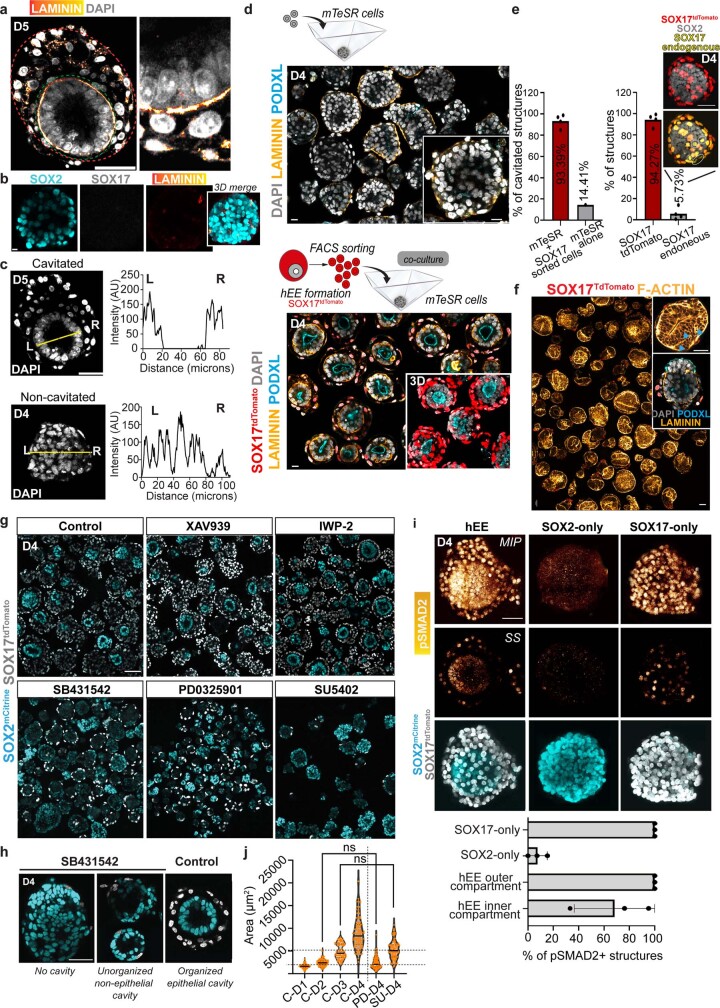
Hypoblast-like specification is required for patterning. (**a**) LAMININ expression between embryonic- (green dotted lines) and extra-embryonic-like (red dotted lines) layers. Scale bar, 50 μm. N = 262 D4-D6 structures, 5 independent experiments. (**b**) A representative SOX2+ aggregate that lacks an extra-embryonic-like layer. Scale bar, 20 μm. N = 262 D4-D6 structures, 5 independent experiments. (**c**) Kymographs of DAPI fluorescence intensity across a representative cavitated hEE and non-cavitated structure that lacks an extra-embryonic-like layer (obtained from mid-z-section). Yellow lines show positions used to plot intensity profiles. (**d**) **Top**: 3D aggregation strategy with primed (mTeSR) hPSCs result in disorganised aggregate formation (wide-field tile scan). Insert image shows a close-up of a disorganised aggregate. **Bottom**: 3D aggregation strategy with wild-type primed (mTeSR) hPSCs and hEE-derived SOX17-tdTomato+ cells result in organised structure formation (wide-field tile scan). Insert image shows organised hEE formation with PODXL+ central cavities. Scale bars, 20 μm. (**e**) **Left**: Percentage of single-cavitated structures in panel d. mTeSR + hEE-derived SOX17-tdTomato+ cell co-culture (N = 439); mTeSR alone (N = 118). Four data points were collected from multiple developmental time points over 2 independent experiments with co-culture trials. The mTeSR-alone group consists of a single time-point from a representative experiment. **Right**: Percentages of structures that exhibit SOX17-tdTomato+ (N = 410/439) or structures with (endogenous) SOX17+ immunolabelled cells (N = 29/439) from the mTeSR + hEE-derived SOX17-tdTomato+ cell co-culture experiment. An example hEE is provided above the bar plot, circled cells lack tdTomato signal, suggesting their endogenous specification from primed hPSCs. Plots show mean ± SD. Four data points were collected from multiple developmental time points over 2 independent experiments with co-culture trials. Scale bar, 50 μm. (**f**) wild-type mTeSR-hPSCs co-aggregated with directed-differentiated SOX17-tdTomato+ definitive endoderm cells generated from primed hPSCs (see Methods). The resulting structures show multiple layers of epiblast-like cells (arrowheads) and high inter-variability between structures. N = 81 aggregates from a representative experiment. Scale bar, 50 μm. (**g**) Wide field tile scans of D4 structures treated with different inhibitors. Scale bars, 100 μm. C–control (N = 220); XAV (N = 101); IWP (N = 132, 2 experiments); SB (N = 125); PD (N = 66); SU (N = 109). The plot shows mean ± SD. 3 independent experiments. (**h**) Phenotype examples of SB431542-treated structures. Scale bar, 50μm. Control (N = 220); SB (N = 125), 3 independent experiments. (**i**) **Top**: Representative D4 hEEs stained with phospho-SMAD2 show active or inactive TGFβ/NODAL in different compartments. Scale bars, 50μm. **Bottom**: Percentage of phospho-SMAD2+ structures. hEE (N = 186), SOX2-only (N = 81), SOX17-only (N = 15). 3 independent experiments. Each dot represents an independent experiment. Plots show mean ± SD. (**j**) Area measurements of hEEs. Each dot represents one structure. The midline (black line) represents the median and the dotted line represents the quartiles within each violin plot. C-D1: Control-D1 (N = 109), C-D2: Control-D2 (N = 86), C-D3: Control-D3 (N = 73), C-D4: Control-D4 (N = 97), PD-D4: PD pre-treated D4 (N = 100), SU-D4: SU pre-treated D4 (N = 94). 3 independent experiments. Two-sided unpaired, parametric t-test with Welsch’s correction. ns, non significant p = 0.6789 for C-D2 vs PD-D4; p = 0.2560 for C-D3 vs SU-D4. Illustrationss in **d**, credit: A. L. Cox. Source Data

Using hEEs, we further investigated the role of outer extra-embryonic hypoblast-like cells as they co-develop and potentially direct morphogenesis within the pluripotent inner compartment. By D3, hEEs contained a laminin-containing basement membrane, which co-occurred with the reorganization of pluripotent epiblast-like inner cells into an apicobasal polarized, central-lumen-containing epithelium (Fig. 3a and Extended Data Fig. 6a). These structural and cell-level morphologies are collectively characteristic of the post-implantation epiblast tissue architecture in the natural embryo^37–39^ and are highly efficient features of hEEs that emerge in 90.71% of all structures examined between D4 and D6.

Our scRNA-seq data indicate that the source of basement membrane deposition is primarily the outer extra-embryonic-like cells (Fig. 3b). Moreover, structures that lack outer extra-embryonic hypoblast specification failed to undergo epithelialization or lumen formation, strengthening a model in which extra-embryonic cells support these critical morphological transitions (Extended Data Fig. 6b,c). To consolidate these findings, we referred back to mTeSR-grown primed hPS cells, which were initially unable to support hEE formation (Extended Data Figs. 1a,b and 6d). To interrogate whether this reflects the lack of endoderm derivatives or basement membrane cues alone, we aggregated primed hPS cells in the presence of 5% Matrigel (an extracellular matrix substitute) or in co-culture with either FACS-sorted hEE SOX17–tdTomato^+^ cells or with directed differentiated definitive endoderm cells^21^ (Fig. 3c and Extended Data Fig. 6d–f). Adding 5% Matrigel alone or with differentiated definitive endoderm cells induced multiple disorganized cavitations per aggregate, as well as a general high aggregate intervariability (Fig. 3c and Extended Data Fig. 6d,f). By contrast, 93.39% of aggregates co-cultured with FACS-sorted hEE hypoblast-like cells demonstrated a polarized epithelium and a centralized lumen within the embryonic compartment (Fig. 3c and Extended Data Fig. 6d,e). These findings confirm an additional essential role of hypoblast cells in patterning the epiblast during implantation.

Finally, by D6, we saw additional transcriptional programming within the hypoblast-like lineage towards a primary (visceral) yolk sac endoderm^14^, including emerging expression of *AFP*, *TTR*, *GJB1*, *VTN* and *APOA2* (Fig. 3d). We also confirmed secreted AFP expression within the outer cells of hEEs as lining an intercellular space (Fig. 3e). These results suggest that the hypoblast-like cells within the hEEs continue towards initial yolk sac-like patterning after their initial role in epiblast-like morphogenesis.

## Inducers of hypoblast-like cell fate

**Extended Data Fig. 7:**
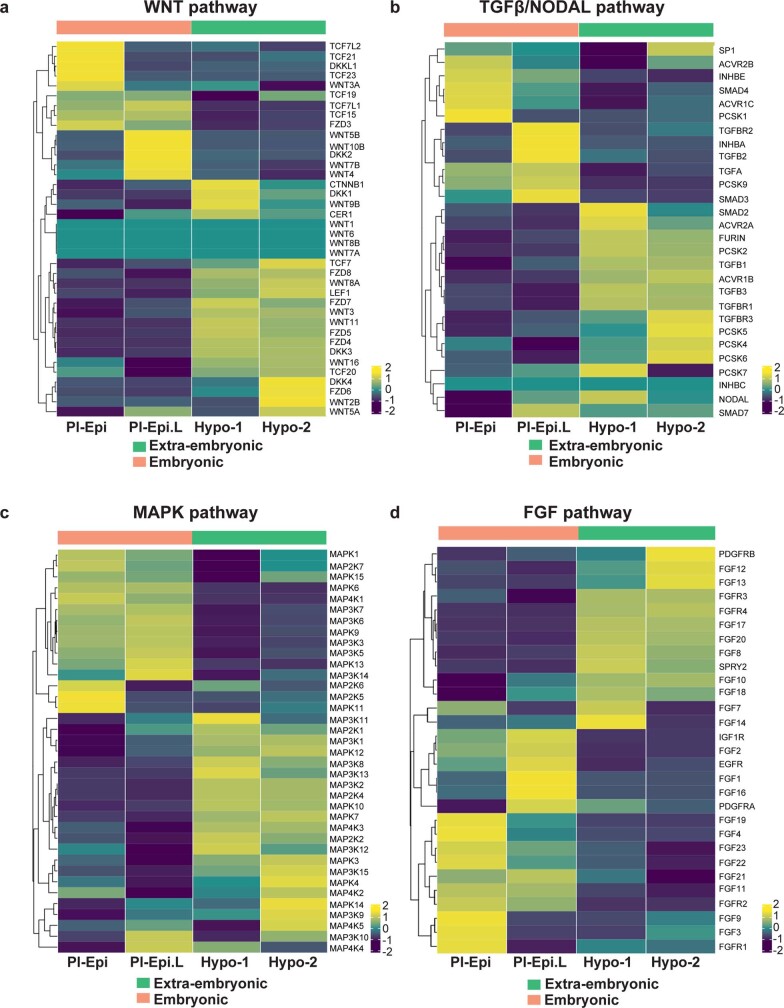
WNT, TGFβ/NODAL, MAPK, and FGF signalling show distinct expression patterns within embryonic- and extraembryonic-like lineages. Expression heatmaps of signalling pathway components in embryonic- (pink) and extra-embryonic-like (green) lineages in the hEE at D4. Normalized expression matrices are row-scaled. Colour scale reflects relative expression level. (**a**) We found that WNT signalling pathway components are widely expressed in both epiblast- and hypoblast-like cells, with particular enrichment of secreted Wnt inhibitors *DKK1*, *DKK3*, and *DKK4* in the Hypoblast-like states. (**b**) TGFβ/NODAL signalling components and transcription factors (*NODAL*, *TGFB1/3*, *SMAD2*, *FURIN*, *PCSK2-7*), as well as receptors (TGFBR1/3 and ACVR1B/2A), also show enriched expression within the Hypoblast-like lineages. (**c**-**d**) MEK and FGF signalling transcription factors are broadly expressed in both epiblast-like and hypoblast-like cells, although the repertoire of signalling pathway components differ between the two cell states. We observed that epiblast-like cells are the source of two major transcripts, *FGF2* and *FGF4* (in panel d), highlighting the similarity to primed pluripotent post-implantation epiblast from human embryos shown before. Of note, analysis of FGF receptors revealed an enrichment of *FGFR3*, *FGFR4*, and *PDGFRB* in hypoblast-like cells, but *FGFR1*, *PDGFRA*, *IGF1R*, and *EGFR* in epiblast-like cells, suggesting differential receptivity to FGF signalling between the two lineages. Postimplantation-Epiblast, PI-Epi-like; Postimplantation-Epiblast Late, PI-Epi.L-like; Hypoblast-1, G1 Hypo-like; Hypoblast-2, G2M/S Hypo-like.

Previous efforts have implicated TGFβ–NODAL, WNT and FGF signalling activity in extra-embryonic endoderm specification from human stem cell cultures and a limited number of human blastocysts^22,40–42^, but these signalling dependencies are still largely unexplored and likely to change as human embryos progress through subsequent developmental stages. Because epiblast-like and hypoblast-like cell fates begin to diverge at approximately 24 h of hEE development, we blocked key signalling axes with small molecules at this time and analysed their effects on multicellular aggregates at D4 (Fig. 3f). SB431542 (a potent TGFβ–NODAL inhibitor), PD0325901 (a MEK inhibitor, downstream of FGFR) or SU5402 (a direct FGFR inhibitor) treatments largely prevented hypoblast-like lineage specification, drastically reducing the number of SOX17^+^ cells and preventing downstream aspects of inner compartment maturation, including polarization and cavity formation (Fig. 3f–h and Extended Data Fig. 6g,h). In support of this result, SOX17^+^ cells expressed phospho-SMAD2 that confirms the activity of the TGFβ–NODAL pathway within the hypoblast-like lineage (Extended Data Fig. 6i), whereas SOX2^+^ cells lack this expression in the absence of outer hypoblast-like cells (Extended Data Fig. 6i). These results further confirm the dependence of the epiblast-like inner compartment on the hypoblast for supporting extracellular matrix components and exogenous signals such as NODAL. Of note, FGF signalling impacts both epiblast-like and hypoblast-like lineages, such that FGF-inhibited aggregates are significantly smaller at D4 and resemble D2 and D3 controls, which confounds our ability to confirm conclusively that the effects of FGF on hypoblast development do not in some way reflect secondary consequences of suppressed epiblast growth (Fig. 3i and Extended Data Fig. 6j). Finally, although XAV939 (a potent WNT signalling inhibitor) treatment had minimal effect on the hypoblast-like lineage, IWP2 (a WNT secretion inhibitor) treatment significantly reduced the complexity of the outer hypoblast-like layer, leading to structures with a ring of single cells surrounding a large SOX2^+^ inner compartment (Fig. 3f,g,i and Extended Data Fig. 6g). Differential expression analysis of major ligands and receptors of the WNT, TGFβ–NODAL and FGF family within D4 hEEs largely supported our findings from our inhibitor treatments, as well as the phenotypes observed in 3D culture (Extended Data Fig. 7a–d). Cumulatively, our data show that robust segregation of human hypoblast-like cells in hEEs primarily depends on TGFβ–NODAL and FGF activity. These results provide insights into how multiple early human lineages co-develop in 3D and how they establish critical crosstalk to guide global morphological rearrangements of these tissues beyond the implantation stages.

## Symmetry breaking by signal antagonism

**Extended Data Fig. 8:**
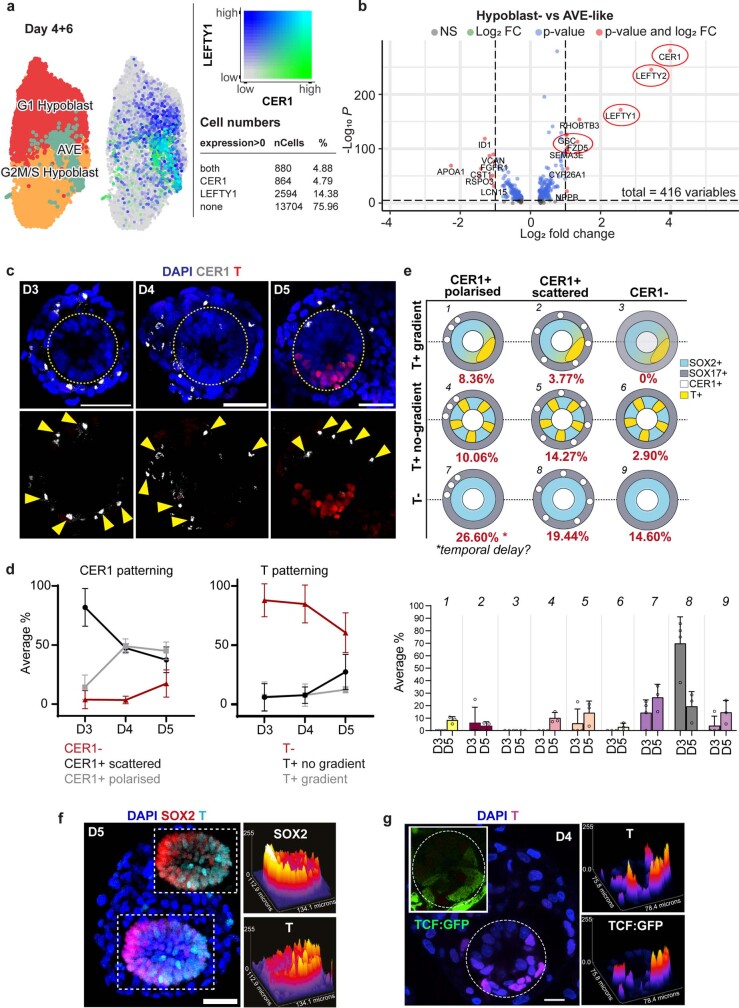
Detailed characterisation of the Anterior Visceral Endoderm-like population in human extra-embryoids. (**a**) **Left**: UMAP of extra-embryonic-like lineages including G1 Hypoblast-like, G2M/S Hypoblast-like, and Anterior Visceral Endoderm-like (AVE) from D4 and D6 hEEs. **Right**: Differential co-expression plots of AVE markers (*CER1* and *LEFTY1*), along with their corresponding percentage and number of cells that express the indicated gene. (**b**) Volcano plot of differentially expressed genes that distinguish AVE-like cells from the rest of the Hypoblast-like lineage. AVE-specific genes *CER1, LEFTY1/2, FZD5*, and *GSC* are highlighted (red circles). A total of 23 genes display significant differential expression (Bonferroni corrected p-value < 1e-05, average Log2 fold change >= 1). Two-tailed non-parametric Wilcoxon Rank-sum test was applied. (**c**) Stack projection of 5–10 mid-sections of DAPI, CER1, and T in D3, D4, and D5 hEEs. Yellow circles enclose the inner, epiblast-like compartment. Yellow arrowheads indicate CER1-positive cells within the hypoblast-like layer. N = 205 structures, 10 independent experiments. Scale bar, 50μm. (**d**) Line graph of CER1 (left) and T (right) expression patterning in D3, D4, and D5 structures. N = 205 structures, 10 independent experiments. Plots show mean ± SD. (**e**) **Top**: Schematic of the phenotypic landscape of CER1 and T expression patterning at D5. N = 99 structures, 3 independent experiments. **Bottom**: Grouped bar graph of average percentages of phenotypes described above in D3 and D5 hEEs (schematics and number codes of phenotypes shown above). N = 128 structures, 7 independent experiments (4 for D3 and 3 for D5). Each dots represent an independent experiment. Plots show mean ± SD. (**f**) Max intensity projection (left) and surface intensity plots (right) of opposing T and SOX2 gradients within the inner compartment (dotted box) in a representative D5 hEE. Scale bar, 50 μm. 12/99 structures show a T+ gradient. 3 independent experiments. (**g**) A single section of TCF/Lef:H2B-GFP and T (left) and surface intensity plots (right) in a representative D4 hEE. The white, dotted circle encloses the inner, epiblast-like compartment. N = 125 structures, 2 independent experiments. Scale bar, 50 μm. Source Data

Beyond ethical limitations and restricted access, many dynamic embryological processes are challenging to study in human without high replicate power or continuous monitoring. We were intrigued by the emergence of a transient transcriptional state within the hypoblast-like lineage that resembled the AVE, an extra-embryonic signalling centre required for specifying anteroposterior patterning in mice^27^ and non-human primates^30,32,43^ (Fig. 2a,b and Extended Data Fig. 3a–c). This subpopulation showed substantial and unique co-expression of classic BMP, NODAL and WNT signalling antagonists, including *CER1*, *LEFTY1*, *GSC* and *LEFTY2* (Extended Data Fig. 8a,b). In addition, *FZD5*, a recently identified primate-specific AVE-associated gene^32^, is also differentially expressed in this subpopulation (Extended Data Fig. 8b). Collectively, the striking co-expression of morphogen antagonists suggested a role for these cells in developmental patterning by attenuating BMP, NODAL and WNT signals to create morphogen gradients.

**Fig. 4:**
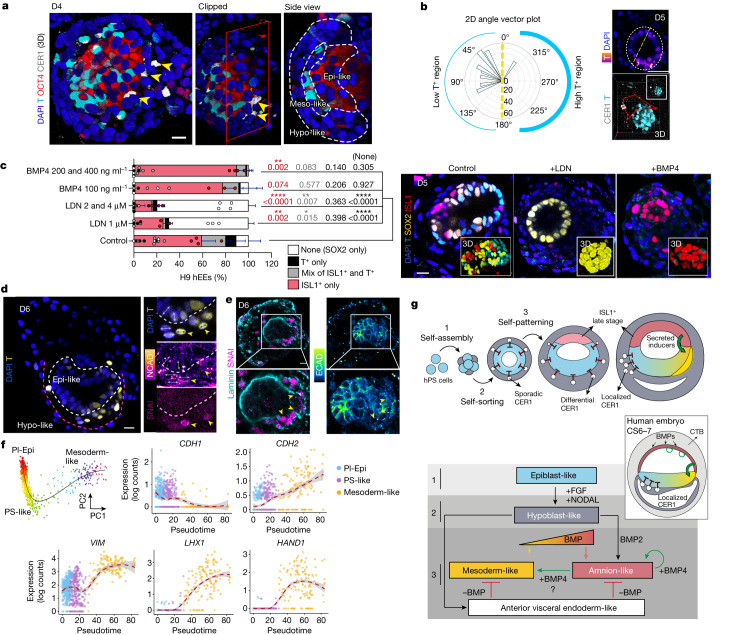
hEEs recapitulate key hallmarks of human perigastrulation. **a**, 3D projections of T, OCT4 and CER1 (arrowheads) expression. The dashed lines demarcate mesodermal-like, epiblast-like and hypoblast-like regions. Note that the CER1^+^ signal in T^+^ cells is expected due to the maturing mesodermal state. *n* = 176 structures, 6 independent experiments. Scale bar, 20 µm. **b**, Angular distribution (left) of CER1^+^ cells relative to T^+^ cells (right; single section on the top and 3D projection on the bottom). Each vector represents the angle for one CER1^+^ cell. The vector length corresponds to the distance between a CER1^+^ cell and the midpoint of the bisecting line (dashed line). Scale bar, 50 µm. **c**, Phenotype frequencies after LDN or BMP4 treatments (left) and the corresponding representative structures (right). Control (*n* = 160), LDN 1 µM (*n* = 64), LDN 2 and 4 µM (*n* = 104), BMP4 100 ng ml^−1^ (*n* = 67) and BMP4 200 and 400 ng ml^−1^ (*n* = 90). Three or more independent experiments (per group) specific to the H9 background are presented in the graph. Six or more total experiments were conducted across different genetic backgrounds. Mean ± s.d. Post-hoc Dunnett’s multiple comparison test, one-way ANOVA. *P* values are shown in the figure. Each dot represents an independent experiment. Scale bar, 20 μm. **d**, T expression in D6 hEE. The zoomed-in images highlight T^+^, NCAD^+^ and SNAI1^+^ cells (arrowheads). The double-headed arrowheads show nuclear reorientation of T^+^ and SNAI1^+^ cells. The white and red dashed lines enclose epiblast-like and hypoblast-like regions, respectively. *n* = 20/53 T^+^ structures, 4 experiments. Scale bar, 20 μm. **e**, SNAI^+^ cells are peripheral to the inner compartment (white box), show downregulated E-cadherin (ECAD), breach the basement membrane (laminin; arrowheads) and focally migrate from the epiblast-like compartment. *n* = 32/55 T^+^ structures, 3 experiments. Scale bar, 20 μm. **f**, Principal curves of hallmark gastrulation markers over pseudotime (post-implantation epiblast-like to primitive streak-like to mesoderm-like states) in hEEs. PC, principal component. Dashed red line indicates PC, grey background indicates LOESS function (locally estimated scatterplot smoothing). **g**, Proposed mechanism of early human development as modelled in hEEs. Also note Fig. 2d for observed spontaneous heterogeneity in the amnion-like specification in hEEs. CTB, cytotrophoblast. Illustrations in **g**, credit: A.L. Cox. Source Data

We confirmed the spatiotemporal emergence of these cells by immunofluorescence. Specifically, the key BMP antagonist CER1 has an initially widespread and scattered distribution across the hypoblast-like compartment before becoming restricted to a distinct region at later time points: 81.81% of D3 hEEs have broadly distributed AVE-like cells, whereas 45.02% of D5 hEEs have polarized distributions (Extended Data Fig. 8c,d). Consistent with our transcriptional data, the overall fraction of CER1^+^ AVE-like cells decreased from D3 to D5, which co-occurs with a rise in the number of mesodermal cell differentiation marker T^+^ (BRACHYURY) cells in the inner compartment (Extended Data Figs. 3a and 8c,d). By D5, 8.36% of structures demonstrated a clear polarized distribution of CER1^+^ and T^+^ (Fig. 4a,b and Extended Data Fig. 8e), and 19.44% of structures with scattered CER1^+^ distribution showed no T induction. These results support the idea that AVE-like antagonism protects the future ectoderm from primitive streak induction. We additionally confirmed that T induction is inversely correlated with SOX2 expression and mirrors critical WNT morphogen gradients via a TCF/Lef:H2B–GFP reporter hPS cell line (Extended Data Fig. 8f,g). Of note, hEEs also induce T in instances in which CER1^+^ cells have no spatial bias (28.10% of all hEEs), which may indicate a degree of independence between the two compartments that requires deeper investigation (Extended Data Fig. 8e). Nonetheless, these data show evidence that the inner and outer compartments co-evolve in vitro, with the outer hypoblast-like layer transiently maintaining AVE-like cells that secrete growth factor antagonists. Although seemingly not sufficient to determine the site of posterior primitive streak induction, these features of symmetry breaking have not been deeply studied outside of rodent embryos^15,27^, highlighting the value of our system for future exploration.

## Progression from AVE to amnion induction

**Extended Data Fig. 9:**
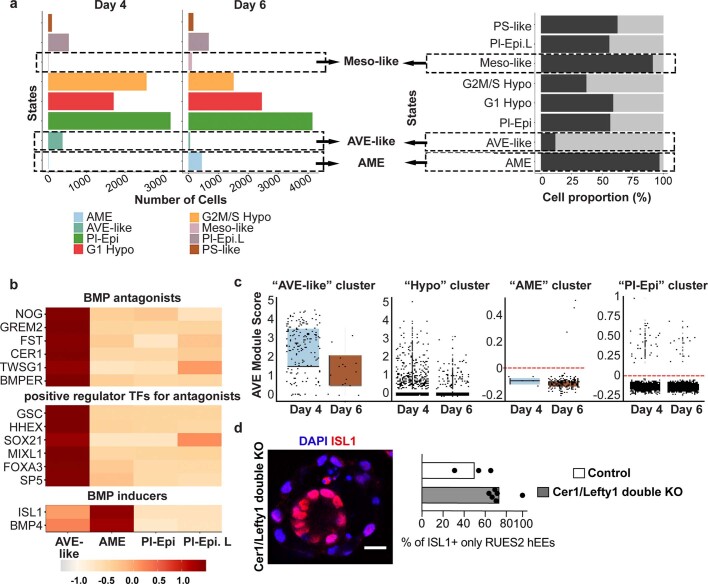
Temporal formation of distinct cell states in human extra-embryoids. (**a**) Bar plots of cell number (left) and proportional representation within D4 and D6 time points (right) per cell state in hEEs. PI-Epi, Postimplantation Epiblast-like (green); AME, Amnionic Ectoderm-like (light blue); PI-Epi.L, Postimplantation Epiblast-Late-like (grey); PS-like, Primitive Streak-like (brown); Meso-like, Mesoderm-like (pink); G1 Hypo, Growth 1 Hypoblast-like (red); G2M/S Hypo, Growth 2 Mitosis/ Synthesis Hypoblast-like (yellow); AVE-like, Anterior Visceral Endoderm-like (teal). (**b**) Heatmap of BMP antagonists (top), positive regulators of transcription factors that induce these antagonists (middle), and BMP inducers (bottom) in hEEs. Normalized expression matrices are row-scaled, colour scale reflects relative expression. (**c**) Temporal expression of AVE gene module scores (*CER1*, *LEFTY1*, *OTX2*, *HESX1*, *NOG*, and *DKK1*) in AVE-like, Hypoblast-like, AME and PI-Epi lineages in the hEEs at D4 and D6. Plots show minimum and maximum values (black, vertical lines) and median (black, horizontal lines). (**d**) Representative D5 hEE generated from CER1;LEFTY1 double knock-out (dKO) hPSCs (N = 61) and control (N = 489). Percentage of ISL1+ hEEs generated from RUES2 (control) and CER1;LEFTY1 dKO RUES2 hPSCs. 2 independent experiments for dKO, 3 independent experiments for control. Plots show the mean and each dot represents the percentage of structures within a tile scan. Scale bars, 20 μm. Source Data

Hypoblast-like cells are unlikely to represent the sole regulator of tissue patterning and organization. Indeed, the amnion has recently been proposed as a second signalling hub for mesoderm formation and gastrulation in primate embryos^34^. As late-state amnion induction is expected to operate through BMP signalling, we cultured hEEs in the presence of the BMP antagonist LDN and found that a significant majority of hEE structures downregulated ISL1 and T (Fig. 4c). By contrast, BMP4 supplementation increases the expression of both markers and expectedly favours ISL1 in a dose-dependent manner (Fig. 4c). Moreover, the high co-expression of *ISL1* and *BMP4* within hEE amnion cells supports the notion that this lineage may emerge to stabilize embryonic mesoderm differentiation within hEEs (Extended Data Figs. 4f,g and 9a). The requirement of BMP4 for mesoderm formation appears to be conserved across species, but in mice stems from the trophoblast-derived extra-embryonic ectoderm^44^, not the amnion. The absence of trophoblast derivatives in our hEE system supports the existence and function of human-specific alternative signalling centres that induce gastrulation patterning in a stepwise manner.

Our scRNA-seq data also show a consistent rise of the amnion subpopulation within hEEs as the AVE-like hypoblast subpopulation dissipates (Fig. 2a and Extended Data Figs. 3a and 9a). This dynamic suggests that ISL1^+^ late-state amnion cells in hEEs most likely emerge after local release from AVE-controlled BMP antagonism, possibly to amplify BMP pathway activity as a second source for secreted ligands (Extended Data Fig. 9b,c). In support of this hypothesis, the hEEs generated from *CER1*;*LEFTY1* double knockout hPS cells produce a higher fraction of ISL1^+^-only structures (Extended Data Fig. 9d). Of note, CER1 has been previously shown to have widespread expression throughout the hypoblast of peri-implantation-stage primate embryos^30,32,45^. On the basis of our result, we surmise that early BMP antagonism may counteract amniogenesis until the appropriate time in human development, after which the amnion is locally induced to consolidate the primitive streak through positive feedback, as suggested in non-human primates^34^.

Overall, these data suggest that hEEs are formed via the timely and balanced action of restrictive and inductive signals, each sourced within distinct extra-embryonic and embryonic subpopulations to act as gatekeepers in the organization of human developmental patterning.

## Hallmarks of human early gastrulation

**Extended Data Fig. 10:**
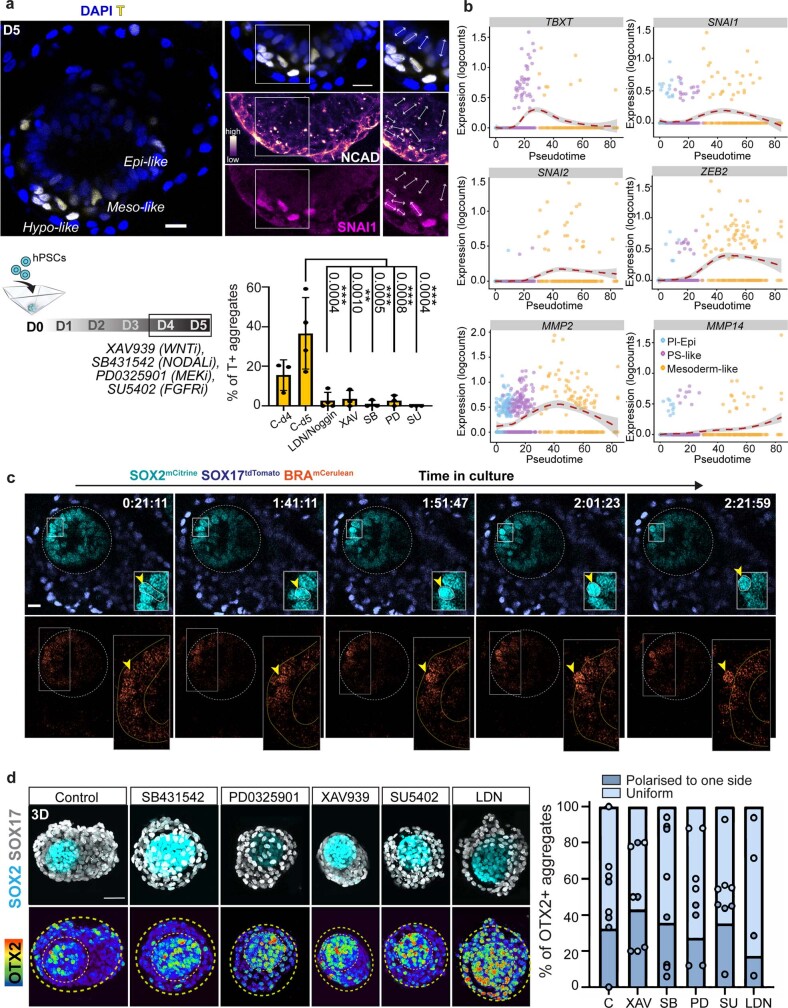
Characteristics of Epithelial-to-mesenchymal (EMT) in hEEs. (**a**) **Top**: T, SNAI1, and NCAD expressions at a representative D5 hEE. The white rectangle indicates ingressed SNAI1+ and NCAD+ cells. Double-headed arrowheads indicate the nuclear orientation of cells. Colour scale represents NCAD intensity gradient. N = 25 structures, 3 independent experiments. Scale bars, 20μm. **Bottom left**: Schematic of growth factors treatments between D4-D5 of hEE development (see Methods). **Bottom right**: Percentage of T+ hEEs. C-D4: Control D4 (N = 176), C-D5: Control D5 (N = 160), LDN (D5) (N = 61), Noggin (D5) (N = 79), XAV (D5) (N = 109), SB (D5) (N = 68), PD (D5) (N = 111), SU (D5) (N = 51). 3 or 4 independent experiments per group. Each dot represents the total percentage of an experiment. Plots show mean ± SD. Post-hoc Dunnett’s multiple comparison test, one-way ANOVA. The figure shows P values. (**b**) Principal curves of hallmark gastrulation markers over pseudo-time (Post-implantation epiblast- to Primitive Streak- to Mesoderm-like states) in hEEs (as shown in Fig. 4f). (**c**) Time-lapse live-imaging of indicated reporter activity. White dotted circles enclose the epiblast-like compartment. Insert image shows an epithelial cell (yellow arrowheads) ingress through EMT. The yellow line shows the organisation of T+ cells. N = 7 hEEs in 2 separate movies. Scale bar, 20 µm. (**d**) **Left**: OTX2 expression of D5 structures from RUES-GLR hPSCs. Yellow, dashed lines represent the embryonic and extra-embryonic compartments. Scale bar, 50 µm. **Right**: Percentage of structures that exhibit different OTX2 expressions within the inner compartment. Control (N = 180), XAV (N = 202), SB (N = 170), PD (N = 224), SU (N = 206), LDN (N = 135). 2 independent experiments for LDN and 4 independent experiments for the rest. Each dot represents an independent experiment. Image in **a**, credit: A. L. Cox. Source Data

After establishing that hypoblast-like and amnion-like lineages are efficiently induced as part of hEE differentiation, we explored their ability to recapitulate early gastrulation dynamics. We identified that by D6, some cell populations that differentiated from an epiblast-like to mesoderm-like state had done so through an epithelial-to-mesenchymal transition (EMT). In particular, we found that asymmetrically localized T^+^ cells downregulate E-cadherin (encoded by *CDH1*) and upregulate N-cadherin (encoded by *CDH2*) and SNAI1 and SNAI2 (hereafter, SNAI), classic molecular markers associated with this process (Fig. 4d,e and Extended Data Fig. 10a). Pseudotime and trajectory inference analysis of our PI-Epi, primitive streak-like and mesoderm-like transcriptional states confirmed that the transcriptional dynamics of epithelial-to-mesenchymal transition are also recapitulated as part of this differentiation process (Fig. 4f and Extended Data Fig. 10b).

Morphologically, E-cadherin^−^N-cadherin^+^SNAI^+^ cells exist in the periphery of the epiblast-like domain and appear to breach the basement membrane as part of a focal migration from the embryonic compartment into the space between it and the hypoblast-like outer later (Fig. 4e). Live imaging of hEEs revealed that these cells also undergo a reorientation in cell shape towards a mesenchymal morphology (Extended Data Fig. 10c; also shown in Fig. 4a,d and Extended Data Fig. 10a). We also subjected D4 structures to 24 h of treatments (to avoid confounding effects of these perturbations on earlier differentiation steps; see Fig. 3) of selective BMP, WNT, NODAL or FGF pathway inhibitors to explore their influence within this window (Extended Data Fig. 10a). All treatment groups significantly blocked T expression, consistent with the collective roles of these pathways in initiating symmetry breaking in mouse and non-human primates^32,46^ (Extended Data Fig. 10a). Conversely, OTX2 expression, an early marker for anterior domains in mouse^47^, did not exhibit any notable responsiveness to inhibitor treatments within the T^−^ inner compartment (Extended Data Fig. 10d). Together, we conclude that the spatiotemporal co-activity of signalling molecules in hEEs leads to subsequent patterning events that mirror both unique and conserved aspects of human perigastrulation.

## Discussion

Here we report a new in vitro approach that efficiently self-assembles to recapitulate key hallmarks of human perigastrulation, including the initial specification of epiblast and hypoblast lineages and subsequent epiblast patterning (Fig. 4g). hEEs achieve these transitions in the absence of trophectodermal cell types, and they represent an ethical opportunity to model the complex interplay between extra-embryonic endoderm and embryonic lineages as they coordinate early human development. Our data demonstrate that to capture the multi-lineage differentiation dynamics in hEEs, the starting hPS cell population must be in the intermediate (formative-to-primed) pluripotency state. As naive pluripotent stem cells have previously been shown to produce blastocyst-stage endoderm progenitors^22^, the limitation of these cells to proceed directly into hEE-like states probably reflects challenges to recapitulating dynamic signalling requirements in vitro, similar to previous observations^19^. The form and function of extra-embryonic endodermal states may change as cells transit through distinct stages of pluripotency, with potential sequential differentiation waves.

Historically, primed hPS cell-derived embryoid bodies permit primitive germ and limited extra-embryonic lineage differentiation, but aggregate assembly and subsequent differentiation are generally chaotic and disorganized, bear little relation to that of the in vivo human embryo, and occur in the absence of an embryo-like shape or patterning^48^ (Supplementary Table 4). By contrast, hEEs offer spatially and temporally organized 3D model generation, allowing researchers to capture multiple complex and interdependent cell-state transitions in a human-specific context. In hEEs, the ISL1^+^ late-state amnion induced by the hypoblast-produced and/or human trophoblast stem cell-produced BMPs lends substantial insights into how the proximodistal axis may form in vivo. However, our current suspension culture protocol leads to a considerably higher ratio of hypoblast-like cells (that expands to surround the inner epiblast-like compartment), which may trigger accelerated signalling and imbalanced lineage representation towards BMP-induced late-state amnion and away from early amnion cell types. We found transcriptional overlap between our in vitro-derived hypoblast-like cells and comparable signatures recovered from human and non-human primate embryos, but epigenetic features that do not deviate considerably from hEPS cells themselves. The seeming inability for hEPS cells to capture the dynamic epigenetic events that co-occur during implantation may reflect an additional feature for future optimization, as could efforts to examine how our current findings relate to the remarkable interconvertibility between embryonic definitive endoderm and extra-embryonic visceral endoderm (VE) lineages that have been identified in mice^24,25,49,50^.

Our hEE system enables careful and dynamic dissection of early embryonic cell types as they shape the early human conceptus. We define the presence of embryonic organizers, from the initial hypoblast to the late-state amnion, and pinpoint key interactions between these cell types as they orchestrate complex aspects of symmetry breaking and morphogenesis. Our results are also consistent with earlier observations made in non-human primate embryos and human stem cell cultures, but enable exploration of human-specific mechanisms through a 3D reconstruction of early human embryonic development. Together, our new platform showcases new opportunities to address unexplored stages of human development in a manner that considers multiple essential parameters, from gene expression to spatial patterning. The ability to reconstitute these processes in vitro may offer new paths for biomedical research that help to overcome scant sample availability and ethical limitations on human embryo research.

## Methods

### Data reporting

No statistical methods were used to predetermine sample size. The experiments were not randomized and the investigators were not blinded to allocation during experiments and outcome assessment.

### Ethics statement

Stem cell-derived multicellular structures described in this study were analysed for and show no evidence of cell types associated with the extra-embryonic trophoblast lineage, which are required for implantation. These multicellular structures do not grow further and are not equivalent to the in vivo human embryo. As they lack trophoblast cell types, they are considered non-integrated embryo models and are not considered as a human embryo according to the International Society of Stem Cell Research (ISSCR). The stem cell research was subject to review and all experiments were approved from the Embryonic Stem Cell Research Oversight Committee (ESCRO) of Yale University, in compliance with the ISSCR 2016 and 2021 guidelines as well as the Connecticut state policies. All experiments on mice were regulated following ethical review by the Yale University Institutional Animal Care and Use Committee. All experiments followed all relevant guidelines and regulations.

### Human cell lines

The hPS cell lines utilized in this study include: RUES2 wild type, RUES2-GLR, RUES2–SMAD1–RFP;H2B–mCitrine^51^, RUES2–CER1;LEFTY1 double knockout RUES2 hPS cells^52^ (provided by A. Brivanlou, The Rockefeller University), ESI017 (ESI BIO), TCF/Lef:H2B–GFP H9 (ref. ^53^) (provided by R. Russe, Stanford University) and H9 (WiCell). Human trophoblast stem cells^26^ (TSCs; TS^CT^) were provided by H. Okae and T. Arima (Tohoku University Graduate School of Medicine). The human endometrial epithelial line was provided by H. Taylor (Yale University)^54^. Each of these cell lines tested negative for mycoplasma contamination, which was monitored on a bimonthly basis (MycoScope PCR Mycoplasma Detection Kit).

### Reproducibility by human cell line genetic variability

Experiments presented in this study were repeated with three different genetic backgrounds of hPS cells (RUES2, ESI017 and H9), over more than six experimental repeats per cell line used. Reproducibility of hEE generation was verified in all hPS cell lines with a yield ranging from approximately 74% to 84% of all aggregates that generated clear embryonic-like and extra-embryonic-like compartments with downstream cavitation and symmetry breaking. It is noted that, under the same experimental conditions, amnion differentiation yields are higher in the H9 background than in the RUES2 and ESI017 backgrounds. In addition, the in vitro developmental timeline can vary ±24 h among different genetic backgrounds of hPS cells (see Supplementary Data Table 2).

### Cell culture

All hPS cell lines were cultured in 37 °C in 5% CO_2_ on mitotically inactivated CF-1 mouse embryonic fibroblasts (MEFs). RSeT hPS cells were cultured in the commercially available medium (STEMCELL Technologies) as per the manufacturer’s instructions, were passaged once domed colonies had formed and medium was changed daily. The hEPS cells were cultured in human expanded potential-complete media (hEP-LCDM media) as previously described^18^, with addition of 1 µM of the WNT inhibitor IWR-1 and 2 µM ROCK inhibitor (Y27632) (hEP-LCDMYI). The naive PXGL hPS cells were cultured as previously described^19^. Human TSCs were cultured in conditions as previously described^26^. All cells were passaged or used for generation of the human extra-embryoid once they reached 80% confluency. Note that no hPS cell line beyond passage number 70 was kept in culture nor used for hEE generation.

### Generation of multicellular aggregates (hEEs) in 3D

The experiment was performed in AggreWell plates^55,56^. The hPS cells were dissociated into single cells with Accutase (Thermo Fisher Scientific) incubation at 37 °C for 3–4 min. Cells were pelleted by centrifugation for 3 min at 1,000 rpm and resuspended in RSeT, hEP-LCDMYI or PXGL media (see above). MEFs were depleted on gelatin-coated plates at 37 °C in 5% CO_2_ on gelatinized tissue-culture-grade plates for approximately 30 min. The cell mixture was then resuspended in spontaneous differentiation medium (SDM). SDM is prepared as follows: 50% of MEF-conditioned medium (see the section ‘MEF-conditioned medium generation for SDM’) and the remaining 50% is added with the medium base, which consists of a 1:1 mixture of DMEM/F12:neurobasal (cat. no. #11330-032), 0.5% N2 supplement, 1% B27 without vitamin A supplement, 1% 100X GlutaMAX, 1% 100X MEM nonessential amino acids, 0.2% 50 mM β-mercaptoethanol (all Gibco), 1% 10,000 U ml^−1^ penicillin–streptomycin (Thermo Fisher Scientific) and 5% knockout serum replacement (Thermo Fisher Scientific).

Single cells resuspended in SDM, counted and added dropwise to the AggreWell 400 format (7,200–12,000 cells total (6–10 cells per microwell) for RSeT; 7,200–12,000 cells total (6–10 cells per microwell) for PXGL-partially capacitated; and 6,000–7,200 cells total (5–6 cells per microwell) for hEPS cell). SDM was supplemented with 5 µM of ROCK inhibitor (Y27632) for the first 24 h of aggregation. On D3 and onwards, the medium was changed to mIVC2 medium (as previously described^15^). The aggregation experiment was performed in 37 °C in 5% CO_2_ and 5% O_2_. We observed that beyond culture D6–D8, multicellular structures did not exhibit spontaneous lumen progression, and overall became disorganized, potentially owing to a lack of supportive environment from other lineages that are not present within this system. Where indicated, 5% Matrigel (Corning) was added to the SDM or mIVC2 media for aggregation of hPS cells.

### MEF-conditioned medium generation for SDM

Two million mitotically inactivated CF-1 MEFs (Gibco) were seeded onto a 100 × 15 mm Petri dish (Corning) and cultured in medium that consisted of: DMEM (cat. no. #11995-040) with 18% inactivated FBS (Gibco), 1.2% 10,000 U ml^−1^ penicillin–streptomycin (Thermo Fisher Scientific), 1.2% 100X Glutamax, 1.2% MEM nonessential amino acids, 1.2% 100 mM sodium pyruvate and 0.24% 50 mM 2-mercaptoethanol (all Gibco). Of conditioned medium, 7–10 ml was collected every 3 days. A maximum of three batches were collected per dish.

### Co-culture with hTSCs and human endometrial cell line

hEEs at D4 or D5 were transferred to 2D human endometrial cells (data shown in Extended Data Fig. 2d) or hTSCs (data shown in Extended Data Fig. 5d) on optic-grade Ibidi plates (Ibidi USA). For this, hEEs growing within the AggreWell were collected by pipetting and transferred to a 35-mm dish where they were individually picked by a Pasteur pipette and transferred onto endometrial or hTSCs. Co-culture was done at 37 °C in 5% O_2_ for 24–48 h and fixed for further processing. For 3D co-culture with hTSCs (data shown in Extended Data Fig. 2b), hTSCs were dissociated into single cells using TypLE express for 7 min at 37 °C followed by neutralization with serum-containing medium. hTSCs were centrifuged for 1 min followed by resuspension in mIVC2 medium. hTSCs were added dropwise onto D5 hEE aggregates in AggreWell 400 (5–10 hTSCs per microwell, 6,000–12,000 total). 3D co-culture was performed for 24 h and fixed the following day for further processing.

### Capacitation (formative pluripotency transition)

Naive PXGL capacitation experiments and medium conditions were performed with a previously published protocol^19^ with slight modifications. In brief, naive PXGL hPS cell colonies were dissociated into single cells with Accutase (Thermo Fisher Scientific) incubation at 37 °C for 3 min. Cells were pelleted by centrifugation for 3 min at 1,000 rpm and resuspended in naive PXGL medium. MEFs were depleted at 37 °C in 5% CO_2_ for approximately 40 min on gelatinized tissue-culture-grade plates. Geltrex-coated plates were prepared with 1:30 Geltrex in DMEM and incubated for 30 min at room temperature followed by 1 h at 37 °C. Next, naive hPS cells were collected and resuspended in naive PXGL medium supplemented with 10 µM ROCK inhibitor (Y27632) and plated on Geltrex-coated plates. After 48 h, hPS cells were washed once with DMEM/F12 supplemented with 0.1% BSA. Capacitation was performed in N2B27-based medium supplemented with the WNT inhibitor XAV939 (2 µM; Sigma-Aldrich) as previously indicated^19^. N2B27-based medium contains 1:1 mixture of DMEM/F12 neurobasal, 10% B27, 5% N2, 10% 100X Glutamax and 1% 50 mM β-mercaptoethanol (all Gibco). The medium was renewed every day. Cells were partially capacitated until D5 before being used for the generation of hEEs as described in the section ‘Generation of multicellular aggregates (human extra-embryoids) in 3D’. The highest potential for the hEE generation from partially capacitated PXGL cells was observed on D5 of the naive-to-primed transition. Beyond D5, this ability rapidly declined similarly to what is observed for conventional primed hPS cells.

### Signal modulation experiments

For functional experiments presented in Figs. 2f, 3f–i, 4c and Extended Data Figs. 6g,h,j and 10a,d, the following treatments were used: 10 µM SB431542 (STEMCELL Technologies), 1 µM XAV-939 (Sigma-Aldrich), 3 µM IWP-2 (Tocris), 10 µM PD0325901 (STEMCELL Technologies), 100 µM SU5402 (R&D systems), 1, 2 or 4 µM LDN (Sigma-Aldrich), 350 ng ml^−1^ Noggin (R&D systems), 100 or 200 ng ml^−1^ recombinant human BMP2 protein (Peprotech), 100, 200 or 400 ng ml^−1^ BMP4 (Fisher Scientific), and 100 or 200 ng ml^−1^ BMP7 (Peprotech). Subsequent medium changes were performed daily with the same concentration in the respective medium.

### FACS

Organized D3 or D4 hEEs were collected by pipetting from AggreWells and washed twice with PBS–0.1% BSA. Following this, hEEs were dissociated into single cells with TryplE incubation at 37 °C for 5 min. After gentle pipetting, TryplE was inhibited with PBS–0.1% BSA and cells were pelleted by centrifugation for 3 min at 1,000 rpm. The single-cell dissociation was confirmed by using a haemocytometer and cells were resuspended in PBS–BSA before being resuspended in FACS buffer and filtered through a mesh into a FACS tube. FACS sorting (S3e Cell Sorter; 1451005, Bio-Rad) was performed to collect SOX2–mCitrine^+^ and SOX17–tdTomato^+^ cells in mIVC2 medium before being used for experiments. For the expansion culture of sorted hEE hypoblast cells (shown in Fig. 1j), a naive primitive endoderm expansion protocol^22^ was applied.

### Co-culture of primed hPS cell aggregation with FACS-sorted hEE hypoblast cells or differentiated definitive endoderm

SOX17–tdTomato^+^ cells were FACS-sorted from D3 or D4 hEEs as described above and primed hES cells (grown under mTeSR conditions (STEMCELL Technologies)) were differentiated to definitive endoderm using a published protocol^21^. Of sorted or differentiated cells, 18,000 cells were mixed with 6,000 hPS cells grown under mTeSR conditions (STEMCELL Technologies) and added dropwise to one well of AggreWell 400. The standard hEE aggregation protocol was followed for both the strategies as described in the section ‘Generation of multicellular aggregates (human extra-embryoids) in 3D’.

### Chimera assay

Mouse embryos at the two-cell stage were recovered from 5–6-week-old CD1 females that were superovulated by injection of 7.5 IU of serum gonadotropin from pregnant mares (ProSpec) followed by 7.5 IU of human chorionic gonadotropin (Sigma) after 48 h and were mated with 8–12-week-old CD1 males. Embryos were recovered in M2 medium (Sigma) by flushing the oviduct. After transferring to KSOM (Millipore), the embryos were cultured in the incubator at 37 °C in 5% CO_2_ for approximately 60 h until they reached early blastocyst stage (E3.25 or E3.5). On the day of injection, D3 or D4 hEEs were collected, washed with PBS and incubated with TrypLE at 37 °C for 5 min. SOX17–tdTomato^+^ cells were sorted via FACS (as described above) and 5–10 cells were microinjected into the mouse early blastocyst cavity and cultured overnight in KSOM at 37 °C in 5% CO_2_ before immunostaining. For postimplantation stages, pseudopregnant surrogates were prepared by mating CD-1 female mice in oestrus with vasectomized Swiss-Webster strain male mice. Approximately 15 injected embryos were transferred to each uterine horn of 2.5-day pseudopregnant surrogates. Surrogates were killed 2 days post-transfer and uteri were recovered for postimplantation embryo dissection from deciduae in a HEPES-buffered medium. The same protocol applied for SOX17–tdTomato^+^ cells derived from primed hPS cells (grown under mTeSR conditions (STEMCELL Technologies)) definitive endoderm differentiation. For definitive endoderm differentiation, we applied a previously published protocol^21^. In our hands, definitive endoderm differentiation efficiency was found around 50–70% as judged by SOX17–tdTomato^+^ induction. All mice were maintained in accordance with national and international guidelines. All experiments have been regulated following ethical review by the Yale University Institutional Animal Care and Use Committee. All experimental mice were maintained in specific pathogen-free conditions on a 12–12-h light–dark cycle temperature-controlled facility with free access to water and food, and used from 5–6 weeks of age. For animal experiments, no randomisation or blinding was performed. 

### Immunofluorescence staining

Immunoflourescence was either performed on bulk or selected groups of multicellular aggregates. Aggregates were fixed in 4% paraformaldehyde for 20 min at room temperature. After fixation, aggregates were washed with 1× PBS–0.05% Tween-20 and permeabilized using 1 mM glycine and 0.3% Triton X-100 in PBS for 20 min at room temperature on a rocking platform. Subsequently, primary antibody incubation was performed overnight at 4 °C in blocking buffer (PBS containing 10% FBS and 10% Tween-20). The next day, the structures were washed with 1× PBS-T, then incubated overnight at 4 °C with secondary antibodies diluted in blocking buffer. On D3, structures were washed with 1× PBS–1% BSA and finally transferred into 1× PBS–1% BSA drops within 30-mm mineral oil-filled (Sigma) glass bottom microwell dishes (MatTek Corporation) before confocal imaging. All antibodies used in this study are listed in Supplementary Table 5.

### Image data acquisition and processing

Samples were imaged with the Leica STELLARIS 5 microscope in tilescan mode, using a HC PL APO CS2 ×40/1.10 water objective, lateral pixel size of 0.569 µm, a Z-spacing of 2–3 µm and appropriate laser and filters for Alexa 405, Alexa 488, Alexa 546 and Alexa 633 or combinations thereof. Single zoomed-in sections were imaged with a Z-spacing of 2 µm and line averaging of 3 for all filters except Alexa 405. The Z-compensation parameters were kept consistent for the respective filters.

### Image analysis

#### Estimates of cell number

Images were acquired with 2-μm Z-separation. Estimated cell numbers within the inner embryonic and outer extra-embryonic compartments were acquired using a Fiji (version 1.53t Java 1.8.0_322 (64-bit)) plugin called Object Scan from the Gurdon Institute (Cambridge, UK). Object Scan generates estimated outlines around individual cells within an aggregate. These outlines were verified manually after each aggregate to ensure each cell was accounted for. The automated cell number estimates from Object Scan were adjusted accordingly after manual verification of cell number. Estimated cell numbers were obtained from the SOX2^+^ inner compartment and SOX17^+^ outer compartment from the surface of the aggregate as deep into the aggregate as signal can still be visualized.

#### Size quantification

Size quantifications of the SOX2^+^ inner compartment and the total aggregate were obtained using Fiji software (version 1.53t Java 1.8.0_322 (64-bit). An outline was drawn around the SOX2^+^ inner compartment and the whole aggregate at the mid-Z-section of a single plane. The area measurement of each outline was acquired for both the SOX2^+^ inner compartment and the total aggregate.

#### CER1 and T expression patterning quantification

For CER1 and T expression patterning quantification presented in Extended Data Fig. 8e, a combination of 3D projection, max intensity projection and single sections on the Leica software (version 3.7.6.25997) were used to determine the spatial relationship between CER1 and T. Only the CER1 signal in the outer hypoblast-like region was considered, as the CER1^+^ signal in T^+^ cells is expected as part of mesodermal differentiation.

#### Angle quantification

For 2D angle quantification presented in Fig. 4b, Leica software (version 3.7.6.25997) was used to quantify the angular distribution of CER1^+^ cells bisecting high and low T^+^ cells in the epiblast-like domain in 2D space. A line was drawn to separate the high from the low T^+^ domains in the epiblast-like region. A vector including the bisecting T^+^ gradient line and one CER1^+^ cell along with the angle measurement of the vector was generated by the software. Finally, the vector plot was generated by the ‘plotly’ package in R studio (R version 4.1.3). For the 3D angle quantification in Fig. 4b, the 3D viewer on Imaris software (Imaris ×64 10.0.0) was used to mark CER1^+^ cells in the hypoblast-like region. CER1^+^ cells were connected to each other using vectors, and angle measurements were taken using the angle measurement function on Imaris software. T^+^ high and low regions were determined using the Fire intensity gradient LUT on Fiji.

#### Diameter measurement

For measuring the diameter of hEEs in Extended Data Fig. 1c, Leica software was used to define the length and height of the mid-Z-plane of each structure. The height axis orientation of each hEE was determined by the shortest distance of the acentric, inner compartment to the outside of the hEE. Once the height axis is assigned, the height axis is the longest distance across the structure. The length axis is the longest distance across the structure perpendicular to the height axis.

#### Image processing

Images were processed in Fiji Image J open access software (1.53t Java 1.8.0_322 (64 bit)) or Leica Application Suite X (3.7.6.25997). Figures 2d,e and 4b,d and Extended Data Fig. 5b,c were processed using the Fire intensity gradient LUT; Figs. 1g and 3a and Extended Data Fig. 6a,i using smart LUT; Figs. 3e and 4e using the Green Fire Blue intensity gradient; Fig. 4d and Extended Data Fig. 10a using the Magma intensity gradient; Extended Data Fig. 6f using Orange hot in Image J Fiji; and Extended Data Fig. 10d using the rainbow gradient spectrum on Leica Las X software. Finally, kymographs in Extended Data Fig. 6c were generated using Fiji (version 1.53t Java 1.8.0_322 (64 bit).

#### SMAD nuclear fluorescence intensity quantification

Nuclear segmentation masks of the epiblast-like compartment of the hEE were generated using Ilastik software (Ilastik-1.4.0). Ilastik was trained to distinguish nuclear from cytoplasmic signal from top, middle and bottom sections for three structures. Once Ilastik could accurately distinguish the cytoplasmic from the nuclear signal, nuclear segmentation masks were generated for the full image sequence of each structure. The nuclear segmentation masks for each section of each structure (approximately 20 sections per structure) was overlaid onto the ISL1 and SMAD channels and the raw integrated density and area measurements were obtained for each nuclear segmentation of the inner compartment using a macro on Fiji. Fluorescence intensity was determined by raw integrated density divided by area. ISL1^+^ and ISL1^−^ cells were distinguished by determining the lowest fluorescence intensity signal in ISL1^+^ analysed cells. All cells above this threshold (600 raw integrated density per area) were classified as ISL1^+^ and all cells below were classified as ISL1^−^.

#### Nuclear circularity measurement

Nuclear circularity measurements of ISL1^+^ only, SOX2^+^ only and ISL1^+^SOX2^+^ cells in Extended Data Fig. 4c were quantified by their respective aspect ratios. The length and height measurements of the mid-section of hEEs were determined using Leica software (version 3.7.6.25997). Height of the nucleus was determined by the longest distance across the nucleus parallel to membrane staining (N-cadherin and F-actin) and perpendicular to the central cavity of the hEE. This axis was also based on the apical and basal orientation of the cell (refer to Extended Data Fig. 4c). The length axis is the longest distance across the nucleus perpendicular to the height axis. ISL1^+^SOX2^+^ were classified by cells with approximately equal fluorescence intensity values for SOX2^+^ and ISL1^+^ signals. Nuclei that were only partially visible on the mid-section of the hEE were excluded from nuclear circularity measurement. Over 70% of cells in the inner compartment in a single plane were accounted for.

### Timelapse live imaging

Confocal timelapse imaging during hEE development, generated from RUES2-GLR hPS cells, was performed using a Leica STELLARIS 5 microscope using a ×10 or ×40 objective and appropriate laser and filters for Alexa 488, Alexa 546 and Alexa 633 or combinations thereof. The structures were imaged at 10–20-min intervals in 40-μm zoomed-in image stacks of 2-μm *z* planes for at least 16 h in on pre-treated Ibidi dishes (Ibidi), under a humidified chamber at 37 °C and 5% CO_2_.

### Single-cell isolation of human extra-embryoids for scRNA-seq

hEEs that exhibited inner and outer compartment organization under brightfield microscopy were collected for single-cell isolation on D4 and D6. These were generated from independent hEPS cell cultures—different clones and passage numbers from the same genetic background (ESI017)—induced into hEEs in two individually prepared AggreWells, for a total of four AggreWells (and two independent experimental replicates) per timepoint. Same timepoint wells were then pooled for disassociation to account for potential batch-level experimental variation in hEE progression. Roughly 200–300 aggregates per timepoint were serially washed through several drops of PBS supplemented with 0.1% BSA before incubation in 200 ml TrypLE (Gibco) at 37 °C for 15 min. Structures were dissociated by pipetting under a stereomicroscope at 5-min intervals and returned to incubation until a predominantly single-cell suspension was confirmed. Although the epithelial cells of the internal compartment dissociated readily, additional time was required to fully dissociate cells in the outside layer due to the appearance of a more extensive extracellular matrix. Once dissociated, cells were collected in 1 ml 0.1% BSA in PBS, filtered using a FlowMi cell strainer (40 mM; Scienceware) and centrifuged for 5 min at 1,000 rpm. After two washes, single-cell suspensions were resuspended at a set volume, counted using a standard haemocytometer and adjusted to 1,000 cells per microlitre. Single cells (*n* = 16,500) for each timepoint were loaded onto a Chromium Controller (10X Genomics) to generate single-cell transcriptomes using the Chromium Next GEM Single Cell 3′ platform according to the manufacturer’s protocol (10X Genomics) and sequenced to more than 300 million read pairs on an Illumina NovaSeq 6000. Before more detailed analyses, the overall runs were validated according to the initial CellRanger outputs, including approximately 30,000 mean transcripts per cell and 3,800 median genes per cell. The overall fraction of transcripts represented by mitochondrial genes was less than 5%. A total of 22,054 cells were obtained for D4 and D6 aggregates. D4 aggregates consisted of 10,826 cells, and D6 aggregates comprised 11,228 cells.

### scRNA-seq data analysis

After initial validation of the library quality, preliminary clustering and filtering steps were taken to identify and eliminate library preparation and sequencing artefacts. These included the removal of cells with more than 10% mitochondrial transcript counts to discard non-viable cells (1,493 cells removed, 6.7% of total), more than 27,500 transcript counts to remove likely doublets (1,069 cells, 4.8% of total) and less than 3,430 transcripts to remove low-quality cells (2,013 cells, 9.0% of total). A total of 19,247 cells were preserved at this stage, corresponding to 85.8% of total cells. Data for D4 and D6 were processed separately using the Seurat (v.4.3.0) package in R (v.4.2.3). Processing steps included library log normalization, data scaling and linear dimensionality reduction through principal component analysis using the 2,000 most highly variable genes in the sample, identified with a variance stabilizing transformation. We used the UMAP algorithm for reduction into two or three dimensions. After calculating cell cycle scores for single cells based on the expression of canonical proliferation markers for S and G2M phases using the CellCycleScoring function in Seurat, we used a regression model to reduce variance derived from cell cycle differences during data scaling, then repeated the dimensionality reduction steps described. All pre-processing was performed with default parameters unless specified. We observed a high degree of separation between D4 and D6 samples in the low-dimensional projection, suggesting potential experimental variation. To address this, we performed data integration using reciprocal principal component analysis (RPCA) to correct technical variance in a conservative manner that aims to preserve timepoint-specific transcriptional states. These integrated data were re-processed as previously described.

For our initial cell-type classification, we used reference label transfer to in vivo embryo data from Xiang et al.^15^, which confidently split hEE data into two populations associated with either epiblast or hypoblast states. hEE cells separate readily into these two compartments, which accounts for the major source of variation within the hEE dataset (extra-embryonic versus embryonic identity was captured in principal component 1 and explains 12.6% of total variance, compared with 1.17% for principal component 2). To perform higher-resolution subclustering, we subset these compartments and subjected them to independent subclustering and quality control of communities with abnormal quality metrics. This final round of quality control preserved 18,042 cells, corresponding to 81.8% of total cells. These subcluster annotations were then used to describe the complete data, as reported in Fig. 2a.

Pseudotime and trajectory inference analyses were performed using Slingshot^57^ (v.2.7.0) for principal curve calculation; SingleCellExperiment^58^ (v.1.12.0) and scater^59^ (v.1.26.1) were used for gene expression visualization over pseudotime (Fig. 4f and Extended Data Fig. 10b). The data were subset to cell types potentially undergoing epithelial-to-mesenchymal transition (PI-Epi, primitive streak-like and mesoderm-like) for this analysis. PI-Epi cells were randomly downsampled for visualization purposes (7,332 down to 387 cells, equivalent to the sum of primitive streak-like and mesoderm-like cells). The principal curve was then traced over the first two principal components to infer pseudo-temporal organization.

We used the AddModuleScore function in Seurat to calculate gene module scores for primitive and definitive endoderm markers (Extended Data Fig. 3g). Gene set enrichment analyses for KEGG pathways^60^ were performed using the WebGestalt^61^ web tool with 10,000 permutations and otherwise default parameters. Gene sets were built using the FindMarkers function in Seurat with test.use = ‘DESeq2’ and otherwise default parameters. Genes with adjusted *P* > 0.05 were discarded. The ShinyApp application was built using ShinyCell6 (v.2.1.0). Violin and bubble plots, gene expression heatmaps and expression UMAPs were generated using ShinyApp. Cell-type and pathway marker heatmaps were plotted with ComplexHeatmaps^62^ (v.2.14.0). The ggplot2 package (v.3.4.2) was used to create boxplots, pie charts and scatterplots. Volcano plots were generated with EnhancedVolcano (v.1.16.0). Cluster markers were identified using Wilcoxon rank-sum with the presto package (v.1.0.0) or the FindAllMarkers function in Seurat.

### Benchmarking against published in vivo embryo datasets

Our initial projection identified two large compartments, consistent with the high degree of variation within the integrated sample that could be explained by principal component 1 (12.6% of total variance by principal component 1 and 1.17% by principal component 2). Of note, the genes contributing to this stratification fit canonical regulators of pluripotent and extra-embryonic endodermal identity (*SOX2*, *POU5F1*, *APOE* and *SERPINE2*). We also performed UMAP reference mapping and label transfer to scRNA-seq data previously generated and described^14,15,28^, which captured human development from 5–11 days post-fertilization and a single sample at approximately 16–19 days post-fertilization, as well as cynomolgus monkey embryos from 11–14, 16 and 17 days post-fertilization, respectively. We first reconstructed these public data using Seurat with parameters as defined by previously published methods^36^. We maintained the reported cell-type assignments of each cell for reconstruction and performed RPCA integration to build a reference dataset with all 50 cell-type labels. To reduce the number of redundant labels, we aggregated similar cell types based on the original annotation and transcriptional similarity assessed through sequential unsupervised clustering (see Extended Data Fig 3e.), settling on 21 cell types (Extended Data Fig. 3d). Using this reduced annotation, we built a logistic regression-based classifier model using CellTypist (v.1.3.0) to transfer reference labels to the hEE cells using the ‘majority_voting’ and ‘best_match’ parameters. We then used the MapQuery function in Seurat to predict the position of hEE cells within the reference UMAP space. The ‘decision score’ in the heatmap of CellTypist scales each single-cell transcriptome and transforms it according to coefficients generated from the logistic regression-based model in CellTypist. Scores were averaged for each hEE cell type and row-scaled to highlight the degree to which they match every reference cell label. We also used the cosine function in the lsa package (v.0.73.3) to assess cosine similarity between hEE and reference cell-state transcriptomes, and ComplexHeatmaps9 (v.2.14.0) to create the associated heatmap. To this end, we used 1,931 of the top 2,000 highly variable genes used to cluster and characterize hEE scRNA-seq data (69 genes did not have a clear match to the cynomolgus data). Rows and columns were clustered in the heatmap using Euclidean distance. For more details on our assignments and analytical approach, see Supplementary Notes 1–4.

### Whole-genome bisulfite sequencing and analysis

Whole-genome bisulfite sequencing was conducted as previously described^24^. In brief, hEPS cell samples were snap frozen and SOX2^+^ and SOX17^+^ fractions of D4 hEEs were purified with FACS using a Sony SH800S Cell Sorter to a minimum of 10,000 cells for each fraction. Cells were then pelleted, snap frozen and subjected to DNA purification using a DNA lysis buffer comprising 10 mM Tris, 10 mM NaCl, 10 mM EDTA, 0.5% SDS and 300 mg ml^−1^ molecular biology grade proteinase K (Roche). After approximately 6 h of incubation at 55 °C, samples were purified via a phenol–chloroform-based phase separation, ethanol precipitation and sheared using a Covaris S220 Ultrasonicator to approximately 400 bp. Re-concentrated DNA was then bisulfite converted using the EZ-DNA methylation Gold Kit (Zymo Research) and synthesized into libraries using the xGen methylation sequencing DNA methylation kit using unique dual index adapters (IDT). Final libraries were amplified with ten PCR cycles and sequenced on a Nova-Seq 6000.

After sequencing, raw reads were subjected to adapter and quality trimming using cutadapt (version 2.4; parameters: --quality-cutoff 20 --overlap 5 --minimum-length 25; Illumina TruSeq adapter clipped from both reads), followed by trimming of 10 and 5 nucleotides from the 5′ and 3′ end of the first read and 15 and 5 nucleotides from the 5′ and 3′ end of the second read. The trimmed reads were aligned to the human hg19 reference genome using BSMAP (version 2.90; parameters: -v 0.1 -s 16 -q 20 -w 100 -S 1 -u -R). A sorted BAM file was obtained and indexed using SAMtools with the ‘sort’ and ‘index’ commands (version 1.10). Duplicates were removed using the ‘MarkDuplicates’ command from GATK (version 4.1.4.1) and default parameters. Methylation rates were called using mcall from the MOABS package (version 1.3.2; default parameters). All analyses were restricted to autosomes and only CpGs covered by at least 5 and at most 150 reads were considered for downstream analyses. hES cell and placental samples were from previously published data: hES cell samples are HUES8 and HUES64 from GSE126958 and the placental samples are CT1 and CT3 from GSE152104. All samples were filtered for a minimum of 10 and a maximum of 150 reads. Correlation between genome-wide CpG methylation levels were calculated using the R function ‘corr’ and visualized using the ComplexHeatmap package. For all other downstream analysis, replicates were averaged after having applied the coverage cut-off. CpG island annotation was downloaded from the UCSC Genome Browser and ‘placenta hyper-CpG islands’ were defined by having a minimum difference of 0.1 between the human placenta and the hES cell averaged sample. Violin plots of 1-kb tiles and CpG islands were calculated by first averaging across regions and then plotting the methylation distribution as a vioplot using the R package ‘vioplot’.

### Statistics and reproducibility

Statistical tests were performed on GraphPad Prism 9.3.1, 9.4.0 and 9.5.1 software. No statistical method was used to predetermine the sample size. Where appropriate, analysis of variance (one-way ANOVA), post-hoc Dunnett’s multiple comparison test or two-sided unpaired, *t*-test with Welch’s correction was applied. All the experiments were performed in at least three biological replicates unless specifically described in the Methods and the figure legends. Figure legends indicate the number of structures and independent experiments performed for each analysis.

### Reporting summary

Further information on research design is available in the Nature Portfolio Reporting Summary linked to this article.

## Online content

Any methods, additional references, Nature Portfolio reporting summaries, source data, extended data, supplementary information, acknowledgements, peer review information; details of author contributions and competing interests; and statements of data and code availability are available at 10.1038/s41586-023-06354-4.

### Supplementary information

Supplementary Note 1 Comparing hEE states to an integrated reference of primate implantation and early gastrulation. Detailed description of single cell RNA sequencing assignments and analytical approach.

Reporting Summary

Supplementary Note 2 Metadata of human extra-embryoids single cell RNA sequencing. Table showing cell type, time point and cell cycle.

Supplementary Note 3 Metadata of integrated reference of three studies of primate development. Table showing datasets that include human and Cynomolgus monkey embryos from Tyser et al. 2021, Xiang et al. 2020, and Ma et al. 2019.

Supplementary Note 4 CellTypist decision scores between human extra-embryoid cells and in vivo reference states of three studies of primate development. Tables show the scores which indicates the similarity level to each respective reference label.

Supplementary Table 1 Cell number and proliferative rates of human extra embryoids and primate embryos. Table details the cell number increase over the course of human extra embryoid development in comparison to primate embryos as reported previously.

Supplementary Table 2 Lineage efficiency of human extra embryoids generated from different genetic background hPSC lines. Table details efficiency per feature as observed in distinct genetic background hPSC lines.

Supplementary Table 3 Cell type annotations from Ma, Tyser, and Xiang datasets. Table defines cell type annotations from reference single cell RNA sequencing datasets.

Supplementary Table 4 Feature comparison between human extra embryoids and embryoid bodies. Table details features observed in human extra embryoids in this study versus embryoid bodies as reported previously.

Supplementary Table 5 List of antibodies used in this study.

### Source data

Source Data Fig. 1

Source Data Fig. 2

Source Data Fig. 3

Source Data Fig. 4

Source Data Extended Data Fig. 1

Source Data Extended Data Fig. 2

Source Data Extended Data Fig. 4

Source Data Extended Data Fig. 5

Source Data Extended Data Fig. 6

Source Data Extended Data Fig. 8

Source Data Extended Data Fig. 9

Source Data Extended Data Fig. 10

## Data Availability

The scRNA-seq data for hEEs generated in this study have been deposited in the Gene Expression Omnibus database under the accession code GSE208195. Published human gastrula in vivo, human post-implantation embryos in vitro and cynomolgus monkey in vitro embryo datasets used in this study were obtained from refs. ^14,15,28^ under accession numbers E-MTAB-9388, GSE136447 and GSE130114, respectively. For whole-genome bisulfite sequencing data, previously published hES cell samples were obtained from GSE126958 and the placental samples were obtained from GSE152104. All other data are available on request. Source data are provided with this paper.

## Code availability

The code generated in this study is provided at https://github.com/Smith-Lab-YSCC/hExtra_embryoid.
