# Supplementary material for: Self-patterning of human stem cells into post-implantation lineages
Source: Nature. 2023 Jun 27;622(7983):574–83. doi: 10.1038/s41586-023-06354-4 (PMC10584676; doi:10.1038/s41586-023-06354-4)
Supplement: Supplementary file 1 — Comparing hEE states to an integrated reference of primate implantation and early gastrulation. Detailed description of single cell RNA sequencing assignments and analytical approach. [file 41586_2023_6354_MOESM1_ESM.docx]

Supplementary Note 1 – Pedroza et al. 2023

**Comparing hEE states to an integrated reference of primate implantation and early gastrulation**

Comparing *in vitro* stem cell-based models of human embryogenesis to native development is challenged by the limited number of reference data sets, which to date frequently include only one or several samples and only investigate limited snapshots. To perform our comparisons, we aggregated single-cell RNA-seq datasets for human and Cynomolgus monkey embryos from Tyser et al. 2021, Xiang et al. 2020, and Ma et al. 2019, which span from late preimplantation and early gastrulation. We included the Cynomolgus data set as pure investigation of human reference points led to a large gap between the period of early implantation (Xiang et al.) to the single Carnegie Stage (CS) 7 stage human gastrulating embryo sampled as part of Tyser et al.

These data were integrated using the Seurat package (v.4.1.1) and the RPCA method. Collectively, this integrated reference was composed of 2,847 cells (Tyser: 1,119 cells, 5-185 cells over 19 States); Xiang: 499, 11-90 cells over 15 states; Ma: 1,229, 14-404 cells over 16 states). Cumulatively, this led to a total of 50 cell states as previously reported in their original publications and integrated as part of Rostovskaya et al., 2022 **(Extended Data Figure 3D)**. The large number of states show a substantial dynamic range in the number of cells that define them, from 5 cells in hsPGC from Tyser et al., to 404 cells in cyEXMC state in Ma et al. Initial efforts to train logistic-regression based models to discriminate across so many states led to ambiguous scoring, particularly given the variable number of cells within these states, their different sequencing technologies, and their often biologically expected transcriptional similarities (for example, between Amniotic and Non-Neural Ectoderm, the clear definitions of which are still being refined).

To circumvent the limitation in current reference data, we elected to merge closely related states according to their transcriptional similarities as well as by their developmental timing and origin. To identify relationships between these original annotations, we performed standard preprocessing steps on our integrated object using the first 30 principal components to compute a nearest neighbor graph, followed by clustering at resolution parameters ranging from 0.2-2 in steps of 0.2 (see GitHub). This approach allowed us to detect cell states that were consistently clustered together at different resolutions, such as human and cynomolgus monkey syncytiotrophoblast (STB) states, hemogenic and myeloid progenitors, as well as certain amniotic and non-neural ectoderm states (AME and NNE). We therefore merged these independent reference states into “STB,” “Blood” and “AME/NNE”. Conversely, we identified states that were frequently convoluted in the clustering, such as Primitive Endoderm (PrE), which was split evenly between a cluster containing Hypoblast and Yolk Sac Endoderm and another containing Definitive Endoderm. This was expected given the transcriptional similarity between definitive and primitive endoderm states. Therefore, we opted to keep PrE, hypoblast, and Definitive Endoderm states separate as independent annotations for use in reference label transfer.

Using this new annotation, we utilized CellTypist (v.1.3.0, Dominguez Conde, C. *et al. Science* 2022) to classify the cells in the hEE single-cell dataset using the following parameters. First, we filtered cells to those expressing at least 200 genes, and removed genes expressed in < 3 cells. Given the technical discrepancies between the reference and query (86,838 genes for 2847 cells in the integrated reference, 24,221 genes for 18,042 cells in the hEE data), we opted to utilize only the 2,000 most highly variable genes from the hEE dataset to train the logistic regression model. Of these, 1907 genes were present in the filtered reference, with the remainders representing unannotated or long-coding RNA genes. We trained the model using our reported annotation and default parameters for CellTypist’s “train” function, then performed label transfer with the “annotate” function using “best match” and “majority voting”. The results of the label transfer are reported in Extended Data Figure 3E. Overall, confidence scores for the assignment were low, consistent with the technical discrepancies between reference and data, and the very low number of available cells for a training reference (CellTypist included models trained on > 50,000 cells). However, CellTypist confidence scores represent the linear combination of genes and model informed coefficients, and our Extended Data Figure 3E confirms that these scores are substantially higher for the best matched assignments between hEE and the reference, to all other possible states. Specifically, majority assignment correctly identified the hEE Epiblast cells as “PostEPI”, AME cells as “AME/NNE”, Mesoderm-like cells as “Emergent Mesoderm”, and Hypoblast and AVE-like cells as primarily “PrE,” with some additional similarity to “Hypoblast” (**Extended Data Figure 3E**). These labels are thus concordant with our original annotations and further confirm the similarity between in vitro derived hEE states and those that emerge during early primate implantation and gastrulation.
