# Supplementary material for: Self-patterning of human stem cells into post-implantation lineages
Source: Nature. 2023 Jun 27;622(7983):574–83. doi: 10.1038/s41586-023-06354-4 (PMC10584676; doi:10.1038/s41586-023-06354-4)
Supplement: Supplementary file 2 — Reporting Summary [file 41586_2023_6354_MOESM2_ESM.pdf]

nature research

Corresponding author(s): Berna Sozen

Last updated by author(s): June 4, 2023

Reporting Summary

Nature Research wishes to improve the reproducibility of the work that we publish. This form provides structure for consistency and transparency in reporting. For further information on Nature Research policies, see our Editorial Policies and the Editorial Policy Checklist.

Statistics

For all statistical analyses, confirm that the following items are present in the figure legend, table legend, main text, or Methods section.

n/a	Confirmed	
☐	☒	The exact sample size (*n*) for each experimental group/condition, given as a discrete number and unit of measurement
☐	☒	A statement on whether measurements were taken from distinct samples or whether the same sample was measured repeatedly
☐	☒	The statistical test(s) used AND whether they are one- or two-sided<br>*Only common tests should be described solely by name; describe more complex techniques in the Methods section.*
☒	☐	A description of all covariates tested
☒	☐	A description of any assumptions or corrections, such as tests of normality and adjustment for multiple comparisons
☐	☒	A full description of the statistical parameters including central tendency (e.g. means) or other basic estimates (e.g. regression coefficient) AND variation (e.g. standard deviation) or associated estimates of uncertainty (e.g. confidence intervals)
☐	☒	For null hypothesis testing, the test statistic (e.g. *F*, *t*, *r*) with confidence intervals, effect sizes, degrees of freedom and *P* value noted<br>*Give P values as exact values whenever suitable.*
☒	☐	For Bayesian analysis, information on the choice of priors and Markov chain Monte Carlo settings
☒	☐	For hierarchical and complex designs, identification of the appropriate level for tests and full reporting of outcomes
☒	☐	Estimates of effect sizes (e.g. Cohen's *d*, Pearson's *r*), indicating how they were calculated

Our web collection on statistics for biologists contains articles on many of the points above.

Software and code

Policy information about availability of computer code

Data collection

Single cell RNA sequencing is performed using 10x Genomic Chromium system
The code generated in this study is provided at https://github.com/Smith-Lab-YSCC/hExtra_embryoid.
For microscopy data, samples were imaged with the Leica STELLARIS 5 microscope in tilescan mode, using a HC PL APO CS2 40x/1.10 water objective.

Data analysis

**Image analysis data:
Fiji Image J open access software (version 1.53t Java 1.8.0_322 (64-bit))
GraphPad Prism (version: 9.3.1, 9.4.0 and 9.5.1)
Leica Application Suite X (version 3.7.6.25997)
Imaris (Iversion: x64 10.0.0)
Rstudio (R version 4.1.3 (2022-03-10).
Ilastik (version 1.4.0)
**Single cell sequencing data:
Seurat (version 4.3.0)
dplyr (version 1.1.1)
monocle3 (version 1.3.1)
plotly (version 4.10.1)
SeuratWrappers (version 0.3.1)
ggplot2 (version 3.4.2)
ComplexHeatmap (version 2.14.0)
viridis (version 0.6.2)
presto (version 1.0.0)
stringr (version 1.5.0)
ggrastr (version 1.0.2)

slingshot (version 2.7.0)
ggbeeswarm (version 0.7.2)
scater (version 1.26.1)
gridExtra (version 2.3.0)
lsa (version 0.73.3)
plyr (version 1.8.8)
EnhancedVolcano (version 1.16.0)
magrittr (version 2.0.3)
scanpy (version 1.9.1)
pandas (version 1.4.1)
R (version 4.2.3)
python (version 3.8.13)
celltypist (version 1.3.0)
SingleCellExperiment4 (version 1.12.0)
ShinyCell6 (v.2.1.0)

For manuscripts utilizing custom algorithms or software that are central to the research but not yet described in published literature, software must be made available to editors and reviewers. We strongly encourage code deposition in a community repository (e.g. GitHub). See the Nature Research guidelines for submitting code & software for further information.

Data

Policy information about availability of data

All manuscripts must include a data availability statement. This statement should provide the following information, where applicable:

- Accession codes, unique identifiers, or web links for publicly available datasets
- A list of figures that have associated raw data
- A description of any restrictions on data availability

The scRNA-seq data for human extra-embryoids generated in this study have been deposited in the GEO database under accession code GSE208195. Published human gastrula in vivo, human post-implantation embryos in vitro, and Cynomolgus monkey in vitro embryo datasets used in this study were obtained from Tyser et al. 2021, Xiang et al. 2020, and Ma et al. 2019 under accession numbers E-MTAB-9388 (processed data available at http://www.human-gastrula.net), GSE136447, and GSE130114 respectively. For WGBS data, previously published hESC samples obtained from GSE126958 and the placenta samples obtained from GSE152104. Source data are provided with this paper. All other data is available upon request.

Field-specific reporting

Please select the one below that is the best fit for your research. If you are not sure, read the appropriate sections before making your selection.

☒ Life sciences ☐ Behavioural & social sciences ☐ Ecological, evolutionary & environmental sciences

For a reference copy of the document with all sections, see nature.com/documents/nr-reporting-summary-flat.pdf

Life sciences study design

All studies must disclose on these points even when the disclosure is negative.

Sample size	No statistical methods were used to predetermine sample size. Sample sizes were based on similar studies performed on stem cell-based platforms modelling embryos (e.g., Sozen et al, Nature Communications, 2021; Kagawa et at Nature 2022; Yu et al. Nature 2021)
Data exclusions	Culture experiments: on principle, data were only excluded for failed experiments, reasons for which included suboptimal culture conditions. Spheroid structures displaying a clear inner and outer compartment were used for single cell RNA sequencing analysis. For data collection in embryonic symmetry breaking occurence, only structures that display an inner, embryonic compartment were counted.
Replication	Each result described in the paper is based on at least two independent biological replicates but very often an experiment is based on more. Figure legends indicate the number of independent experiments performed in each analysis.
Randomization	For experiments were our samples were exposed to chemical inhibitors, samples were randomly allocated to control and experimental groups.
Blinding	The majority of the experiments performed are descriptive, and therefore samples were not assigned to experimental groups. For inhibitor treatment experiments, control untreated and experimental treated groups were analysed. In the inhibitor treated, and knock-out groups, blinding was not possible, as inhibitor-treated/KO structures were easily distinguished based on morphological criteria. For all groups, healthy samples of representative stages and status were allocated to ensure comparability.

Reporting for specific materials, systems and methods

We require information from authors about some types of materials, experimental systems and methods used in many studies. Here, indicate whether each material, system or method listed is relevant to your study. If you are not sure if a list item applies to your research, read the appropriate section before selecting a response.

Materials & experimental systems

n/a	Involved in the study
☐	☒ Antibodies
☐	☒ Eukaryotic cell lines
☒	☐ Palaeontology and archaeology
☐	☒ Animals and other organisms
☒	☐ Human research participants
☒	☐ Clinical data
☒	☐ Dual use research of concern

Methods

n/a	Involved in the study
☒	☐ ChIP-seq
☒	☐ Flow cytometry
☒	☐ MRI-based neuroimaging

Antibodies

Antibodies used

alpha-Fetoprotein (AFP) Mouse 1:200 (clone 189502) MAB1368-SP R&D Systems
CERBERUS1 Goat 1:100 (clone DR9046658) AF1075-SP R&D Systems
E-Cadherin Mouse 1:200 (clone 9301695) 610181 BD Biosciences
FOXA2/HNF3 Rabbit 1:300 (clone 3) 8186S Cell Signaling
GABRP Rabbit 1:300, 1:500 (clone XK3759183) PA5-46830 Invitrogen
GATA3 Goat 1:200 (clone UZQ0219011) AF2605 R&D Systems
anti-GFP Rat 1:1000 (clone M8E4658) 04404-84 Nacalai Tesque Inc.
GM130 Mouse 1:100 (clone 7069938) 610822 BD Biosciences
hCG beta Mouse 1:400 (clone MG3298020) MA135020 Invitrogen
HLA-G Mouse 1:100 (clone WG3318857) MA1-19219 Invitrogen
ISLET1 Goat 1:200 (clone JWU0420121) AF1837-SP R&D Systems
LAMININ Rabbit 1:500 (clone WF3299348) PA1-16730 Invitrogen
LEFTY1 Goat 1:100 (clone CMM022201A) AF746 R&D Systems
N-Cadherin Mouse 1:500 (clone WF3303042E) MA1-91128 Invitrogen
Nuclear Antigen (Human) Mouse 1:200 (clone WF3292841) MA5-33098 Invitrogen
OCT4 Mouse 1:200 (clone F0220) sc-5279 Santa Cruz Bio.
OTX2 Goat 1:200 (clone KNO0918121) AF1979 R&D Systems
Phospho-Smad1/5/9 Rabbit 1:800 (clone 10) 9516T Cell Signaling
Phospho-SMAD2 Rabbit 1:800 (clone 3) 18338T Cell Signaling
PODXL Mouse 1:200 (clone JKW0219011) MAB1658 R&D Systems
SNAI1 Goat 1:100 (clone XRS0319091) AF3639 R&D Systems
SOX2 Rat 1:600 (clone 2357438) 14-9811-82 Invitrogen
T/Brachyury Goat 1:200 (clone KQP0719121) AF2085 R&D Systems
T/Brachyury Rabbit 1:250 (clone 1) 81694S Cell Signaling
TFAP2A/AP-2α Mouse 1:300 (clone F0922) sc-12726 Santa Cruz
DAPI 405 1:500 2179275 D3571 Invitrogen
F-Actin (647) 647 1:200 2170291 A22287 Invitrogen
F-Actin (488) 488 1:500 2129460 A12379 Invitrogen
donkey anti-mouse Alexa Fluor 488 1:500 (clone 2147618) A21202 Invitrogen
donkey anti-rabbit Alexa Fluor 488 1:500 (clone 2156521) A21206 Invitrogen
donkey anti-rat Alexa Fluor 488 1:500 (clone 2180272) A21208 Invitrogen
donkey anti-mouse Alexa Fluor 568 1:500 (clone 2110843 A10039 Invitrogen
donkey anti-goat Alexa Fluor 568 1:500 (clone 2160061) A11057 Invitrogen
donkey anti-rabbit Alexa Fluor 568 1:500 (clone 2136776) A10042 Invitrogen
donkey anti-mouse Alexa Fluor 647 1:500 (clone 2136787) A31571 Invitrogen
donkey anti-goat Alexa Fluor 647 1:500 (clone 2175459) A21447 Invitrogen
donkey anti-rabbit Alexa Fluor 647 1:500 (clone 2420695) A31573 Invitrogen

Validation

The subcellular localization of all the proteins analyzed in this study has been previously reported in mouse embryo/stem cell studies (shown as immunoflourescence labelling).

Validation statements available from manufacturers:
anti-AFP: https://www.rndsystems.com/products/human-mouse-alpha-fetoprotein-afp-antibody-189502_mab1368
anti-CERBERUS1: https://www.rndsystems.com/products/human-cerberus-1-antibody_af1075
anti-ECADHERIN: https://www.bdbiosciences.com/en-eu/products/reagents/microscopy-imaging-reagents/immunofluorescence-reagents/purified-mouse-anti-e-cadherin.610181
anti-FOXA2: https://www.cellsignal.com/products/primary-antibodies/foxa2-hnf3b-d56d6-xp-rabbit-mab/8186
anti-GABRP https://www.thermofisher.com/antibody/product/GABRP-Antibody-Polyclonal/PA5-46830
anti-GATA3: https://www.rndsystems.com/products/human-gata-3-antibody_af2605
anti-GFP: https://www.nacalaiusa.com/products/view/101/anti-gfp-rat-igg2a-monoclonal-gf090r
anti-GM130: https://www.bdbiosciences.com/en-us/products/reagents/microscopy-imaging-reagents/immunofluorescence-reagents/purified-mouse-anti-gm130.610822
anti-hCG BETA: https://www.thermofisher.com/antibody/product/hCG-beta-Antibody-clone-5H4-E2-Monoclonal/MA1-35020
anti-HLA-G: http s://www.thermofisher.com/antibody/product/HLA-G-Antibody-clone-MEM-G-1-Monoclonal/MA1-19219
anti ISLET1: https://www.rndsystems.com/products/human-islet-1-antibody_af1837
anti-LAMININ: thermofisher.com/antibody/product/Laminin-Antibody-Polyclonal/PA1-16730
anti-LEFTY1: https://www.rndsystems.com/products/human-mouse-lefty-antibody_af746
anti-NCADHERIN: https://www.thermofisher.com/antibody/product/N-cadherin-Antibody-clone-CH-19-Monoclonal/MA1-91128

anti-OCT4: https://www.scbt.com/p/oct-3-4-antibody-c-10
anti-OTX2: https://www.rndsystems.com/products/human-otx2-antibody_af1979
anti-Phospho-Smad1/5/9: https://www.cellsignal.com/products/primary-antibodies/phospho-smad1-5-ser463-465-41d10-rabbit-mab/9516
anti-Phospho-SMAD2: https://www.cellsignal.com/products/primary-antibodies/phospho-smad2-ser465-ser467-e8f3r-rabbit-mab/18338
anti-PODXL: https://www.rndsystems.com/products/human-podocalyxin-antibody-222328_mab1658
anti-SNAI1: https://www.rndsystems.com/products/human-snail-antibody_af3639
anti-SOX2: https://www.thermofisher.com/antibody/product/SOX2-Antibody-clone-Btjce-Monoclonal/14-9811-82
anti-T/BRACHYURY: https://www.rndsystems.com/products/human-mouse-brachyury-antibody_af2085
anti-T/BRACHYURY: https://www.cellsignal.com/products/primary-antibodies/brachyury-d2z3j-rabbit-mab/81694
TFAP2A/AP-2α https://www.scbt.com/p/ap-2alpha-antibody-3b5
DAPI: https://www.thermofisher.com/order/catalog/product/D3571
F-Actin (647): https://www.thermofisher.com/order/catalog/product/A22287
F-Actin (488): https://www.thermofisher.com/order/catalog/product/A12379
donkey anti-mouse Alexa Fluor 488: https://www.thermofisher.com/antibody/product/Donkey-anti-Mouse-IgG-H-L-Highly-Cross-Adsorbed-Secondary-Antibody-Polyclonal/A-21202
donkey anti-rabbit Alexa Fluor 488: https://www.thermofisher.com/antibody/product/Donkey-anti-Rabbit-IgG-H-L-Highly-Cross-Adsorbed-Secondary-Antibody-Polyclonal/A-21206
donkey anti-rat Alexa Fluor 488: https://www.thermofisher.com/antibody/product/Donkey-anti-Rat-IgG-H-L-Highly-Cross-Adsorbed-Secondary-Antibody-Polyclonal/A-21208
donkey anti-mouse Alexa Fluor 568: https://www.thermofisher.com/antibody/product/Donkey-anti-Rabbit-IgG-H-L-Highly-Cross-Adsorbed-Secondary-Antibody-Polyclonal/A10039
donkey anti-goat Alexa Fluor 568: https://www.thermofisher.com/antibody/product/Donkey-anti-Goat-IgG-H-L-Cross-Adsorbed-Secondary-Antibody-Polyclonal/A-11057
donkey anti-rabbit Alexa Fluor 568: https://www.thermofisher.com/antibody/product/Donkey-anti-Rabbit-IgG-H-L-Highly-Cross-Adsorbed-Secondary-Antibody-Polyclonal/A10042
donkey anti-mouse Alexa Fluor 647: https://www.thermofisher.com/antibody/product/Donkey-anti-Mouse-IgG-H-L-Highly-Cross-Adsorbed-Secondary-Antibody-Polyclonal/A-31571
donkey anti-goat Alexa Fluor 647: https://www.thermofisher.com/antibody/product/Donkey-anti-Goat-IgG-H-L-Cross-Adsorbed-Secondary-Antibody-Polyclonal/A-21447
donkey anti-rabbit Alexa Fluor 647: https://www.thermofisher.com/antibody/product/Donkey-anti-Rabbit-IgG-H-L-Highly-Cross-Adsorbed-Secondary-Antibody-Polyclonal/A-31573

Eukaryotic cell lines

Policy information about cell lines

Cell line source(s)	The hPSC lines utilised in this study include: RUES2 wild type, RUES2-GLR, RUES2-SMAD1-RFP; H2B-mCitrine, CER1/LEFTY1 double knock out RUES2 hPSCs (kindly provided by Ali Brivanlou, The Rockefeller University, US), ESI017 (ESI BIO), TCF/Lef:H2B-GFP H9 (kindly provided by Roeland Russe, Stanford University, US), and H9 wild type (WiCell, US). Human trophoblast stem cells (TSCs; TSCT) were kindly provided by Hiroaki Okae and Takahiro Arima (Tohoku University Graduate School of Medicine, Japan). Human endometrial epithelial line is kindly provided by Hugh Taylor (Yale University).
Authentication	Cells were maintained in conditions to preserve stem cell character and prevent differentiation. Plates were inspected for morphological evidence of differentiation (altered colony morphology in PSC cultures...etc) and plates with differentiated cells were discarded. Furthermore, cell identities were confirmed routinely by immunoflourescence marker expressions.
Mycoplasma contamination	Each of these cell lines was tested negative for mycoplasma contamination, which was monitored and confirmed negative on a bi-monthly basis (MycoScope™ PCR Mycoplasma Detection Kit, Genlantis).
Commonly misidentified lines (See ICLAC register)	The cells we used are not part of this database

Animals and other organisms

Policy information about studies involving animals; ARRIVE guidelines recommended for reporting animal research

Laboratory animals	5-6 weeks old CD1 females and males
Wild animals	the study did not involve wild animals
Field-collected samples	All experimental mice were maintained in specific pathogen-free conditions on a 12–12 -hr light-dark cycle temperature-controlled facility with free access to water and food.
Ethics oversight	All mice were maintained in accordance with national and international guidelines. All experiments have been regulated following ethical review by the Yale University Institutional Animal Care and Use Committee (IACUC).

Note that full information on the approval of the study protocol must also be provided in the manuscript.
